# A Comprehensive Survey of Phenolic Constituents Reported in Monofloral Honeys around the Globe

**DOI:** 10.3390/foods11081152

**Published:** 2022-04-15

**Authors:** Ivan Lozada Lawag, Lee-Yong Lim, Ranee Joshi, Katherine A. Hammer, Cornelia Locher

**Affiliations:** 1 Cooperative Research Centre for Honey Bee Products Limited (CRC HBP), University of Western Australia, Crawley, WA 6009, Australia; ivan.lawag@research.uwa.edu.au (I.L.L.); katherine.hammer@uwa.edu.au (K.A.H.); 2 Division of Pharmacy, School of Allied Health, University of Western Australia, Crawley, WA 6009, Australia; lee.lim@uwa.edu.au; 3 Centre for Exploration Targeting, School of Earth Sciences, University of Western Australia, Crawley, WA 6009, Australia; ranee.joshi@research.uwa.edu.au; 4 School of Biomedical Sciences, University of Western Australia, Crawley, WA 6009, Australia

**Keywords:** honey, monofloral honey, phenolic compounds, polyphenol, flavonoids, hydroxycinnamic acid and derivatives, hydroxybenzoic acid and derivatives, hydroxyphenylacetic acid and derivatives, phytochemistry, biomarkers

## Abstract

The aim of this review is to provide a comprehensive overview of the large variety of phenolic compounds that have to date been identified in a wide range of monofloral honeys found globally. The collated information is structured along several themes, including the botanical family and genus of the monofloral honeys for which phenolic constituents have been reported, the chemical classes the phenolic compounds can be attributed to, and the analytical method employed in compound determination as well as countries with a particular research focus on phenolic honey constituents. This review covers 130 research papers that detail the phenolic constituents of a total of 556 monofloral honeys. Based on the findings of this review, it can be concluded that most of these honeys belong to the Myrtaceae and Fabaceae families and that Robinia (*Robinia pseudoacacia*, Fabaceae), Manuka (*Leptospermum scoparium*, Myrtaceae), and Chestnut (*Castanea* sp., Fagaceae) honeys are to date the most studied honeys for phenolic compound determination. China, Italy, and Turkey are the major honey phenolic research hubs. To date, 161 individual phenolic compounds belonging to five major compound groups have been reported, with caffeic acid, gallic acid, ferulic acid and quercetin being the most widely reported among them. HPLC with photodiode array detection appears to be the most popular method for chemical structure identification.

## 1. Introduction

Honey is a stored food of honeybees (*Apis mellifera*) that originates from plant nectar and is converted to honey with the aid of enzymes secreted from the glands of worker bees. Inside a colony, forager bees with full honey sacs transfer nectar into honeycombs and then flutter their wings to hasten the decrease in nectar moisture before worker bees seal the cells for storage [1]. Honeys are classified either as monofloral/unifloral or polyfloral/multifloral, the former being derived from a predominant botanical species, thus from mainly one type of nectar with only minor, if any, nectar contributions from other botanical sources. Polyfloral honeys, on the other hand, are linked to several botanical sources, none of which predominate [2]. 

Honey has been extensively used throughout history, not only as a food and food sweetener but also for medicinal purposes, which are associated, for example, with its antimicrobial and/or antioxidant properties [2,3,4,5]. However, honey’s potential health benefits can vary considerably due to the diversity of nectar collected by bees as they move from plant to plant [6]. Thus, different phytochemicals present in the nectar of melliferous plants contribute to the variability in honeys’ secondary metabolite profiles and this might also impact their bioactivity levels [3]. 

For bees, honey provides a rich source of carbohydrates, which is reflected in its chemical composition of at least 60% glucose and fructose combined, approximately 10% other sugar constituents and approximately 18% water. The remaining 2–3% of honey consists of a diverse mixture of more than 200 individual compounds including carotenoids, flavonoids and phenolics, along with several other minor components, such as proteins, free amino acids, minerals, vitamins and organic acids [7,8]. These minor constituents are considered to be very important in influencing not only the organoleptic characteristics of honeys but also their respective bioactivity profiles [3]. 

The term ‘phenolic’ or ‘polyphenol’ is chemically defined as a substance that possesses an aromatic ring bearing one or more hydroxyl substituents including functional derivatives such as esters and glycosides. These compounds, which can be further divided into subgroups such as phenolic acids and flavonoids, are extensively found across the plant kingdom and are closely linked to the sensory and nutritional quality of fresh and processed plant foods. Within the phenolic acid subgroup, hydroxybenzoic acid (such as methyl syringate, gallic acid, ellagic acid, protocatechuic acid, syringic acid, benzoic acid and 4-hydroxybenzoic acid), hydroxycinnamic acid (such as chlorogenic, vanillic, caffeic, p-coumaric and ferulic acids) and hydroxyphenylacetic acid (such as homogentisic and phenylacetic acids) derivatives have been detected in various honey samples around the world. Among the flavonoid groups, flavonols (such as myricetin, kaempferol, 8-methoxy kaempferol, quercetin, isorhamnetin, quercetin-3-methyl ether, quercetin-3,7-dimethyl ether, pinobanksin, rutin and galangin), flavones (such as genkwanin, luteolin, apigenin, tricetin and chrysin) and flavanones (such as pinocembrin and pinostrobin) have also been identified in some honeys [6]. 

Despite their relatively minor presence, phenolic compounds are one of the most studied honey constituents due to their well-known biological activities [3,9]. They are, furthermore, reported to influence the organoleptic characteristics of honeys [3] and can also potentially be used to identify or confirm the botanical origin of honeys [9]. Most of the floral markers in honey, which are derived from the nectar of melliferous plants, are flavonoids or phenolic acids. The identification of these compounds in honey can, thus, be an important tool for the recognition of its floral type [9]. Furthermore, phenolic compounds can also be used to monitor honey quality in order to choose the best processing practices [10]. 

A number of reviews have already been carried out on honey phenolics. These reviews can be categorized into honey phenolic analysis, determination and separation [11,12,13,14]; phenolics as authentication and marker compounds for the botanical origin of honey [2,15,16,17]; and honey phenolics and their associated health benefits [3,18,19,20,21,22]. A drawback of these reviews is that they tend to focus only on a few subsets of monofloral honeys found in particular regions. To our knowledge, a comprehensive review of all phenolic compounds isolated to date from honeys around the globe has not yet been published and is therefore the subject of this study. This paper, thus, presents a comprehensive survey of phenolic compounds reported across a very wide range of monofloral honeys from different geographical locations worldwide to provide an overview of their respective phenolic profiles and to assist with the identification of ubiquitous phenolics that are found across various floral sources and across different geographical locations. It also provides information on the botanical orgins of honeys for which phenolic constituents have to date been identified and allows determination of regional hotspots of research on phenolic honey constituents. 

## 2. Materials and Methods

### 2.1. Literature Search

To ensure that a wide range of publications on phenolics and polyphenols present in honey are captured in this review, a thorough literature search was carried out by the Scopus database (published until January 2021) using combinations of the following sets of keywords: honey phenolic profile, monofloral honey and chemistry, honey and phytochemi*, honey and flavonoid* and honey phenol*. Only papers that were written in English and that reported on phenolic compounds in ripe (thus excluding nectar or unripe honey) monofloral honeys of nectar and honeydew origin produced by *Apis mellifera* bees were included in this review. Google Scholar was also used to further enhance the results of this initial literature search.

### 2.2. Data Tabulation and Representation

Information such as the common name, genus and/or species name, and the family of the monofloral honeys’ botanical source (verified using https://www.gbif.org accessed on 19 January–1 February 2022), country of origin, country where the research was performed, as well as the total number of analysed samples, the detected compounds, and the methods of detection/identification employed in compound determination were recorded and tabulated. The collated information was then grouped based on genus, botanical species and/or common name, and also based on the country where the research was performed.

Guided by pre-existing chemical classifications [23,24,25], the reported compounds were grouped into five classes, namely flavonoids, hydroxycinnamic acid derivatives (HCAD), hydroxybenzoic acid derivatives (HBAD), and miscellaneous/other phenolics as well as non-phenolic compounds. Furthermore, a CAS Registry Number (CAS No.) was assigned to each compound, and synonyms, molecular formula, molar mass, and Simplified Molecular Input Line Entry System (SMILES) information were also determined. Molecular structures were generated based on the SMILES information using ChemDraw version 20.0.0.41, PerkinElmer Informatics, Inc.

The prevalence of studies on phenolic constituents of honeys on a global level as well as in various geographical regions was visually represented using maps generated by ARCGIS Version 10.8. Redlands, CA, USA.

## 3. Results and Discussion

### 3.1. Botanical Classification

The literature search yielded a total of 130 original research articles that detail phenolic compounds identified in monofloral honeys in various countries around the globe. Since most studies analysed more than one monofloral honey, this review captures the published data for a total of 556 monofloral honeys. Their predominant botanical sources can be attributed to a total of 51 plant families; 90 of the reported monoflorals originated from members of the Fabaceae family, 88 from Myrtaceae, 56 from Lamiaceae, 41 from Ericaceae, 34 from Rutaceae, 33 from Fagaceae and 30 from Asteraceae. Thus, these seven plant families appear to be the most common botanical sources of honeys for which phenolic constituents have to date been reported in the literature. 

The reviewed reports were further categorised into 159 monofloral groups taking into account not only the botanical family, but also the common name and/or the genus or species of the honeys’ botanical origin. It was found that 23 of these monofloral groups belonged to the Myrtaceae family, 17 were Fabaceae, 14 Lamiaceae, 12 Asteraceae and 9 belonged to the Rosaceae family. 

It could also be determined from this grouping that Robinia honey (*Robinia pseudoacacia*, Fabaceae), which has been reported in 36 research papers, is the most studied honey with respect to phenolic constituents. This is followed by Manuka honey (*Leptospermum scoparium*, Myrtaceae) with 29 research papers, Chestnut honey (*Castanea* sp., Fagaceae) with 28, Linden honey (*Tilia* sp., Malvaceae) with 25, Rape honey (*Brassica* sp., Brassicaceae) and Heather honey (*Calluna vulgaris* and *Erica* sp. (L.) *Hull,* Ericaceae) with 24 each, Eucalyptus honey (*Eucalyptus* sp., Myrtaceae) with 21, Thyme honey (*Thymus* sp., Lamiaceae) and Buckwheat honey (*Fagopyrum* sp., Polygonaceae) with 16 reports each, and Sunflower honey (*Helianthus* sp., Asteraceae) with 15 reports on phenolic profiling. This grouping, based on the botanical origin of the honeys, was also used to structure the overview on phenolic compounds identified in the monofloral honeys (Table 1). The collated information was further used to create two groups of monofloral honeys—the first group containing high-frequency monoflorals (HFMs), where there are four or more studies reporting on their phenolic constituents (31 monofloral honey groups), and the second group, referred to as other monoflorals (OMs), having three or less studies dedicated to their phenolic composition (128 monofloral honey groups). To date, worldwide research efforts on phenolic constituents have mainly focused on the 31 monofloral honey groups referred to as HFMs in this review. The identification of the HFMs was also used in the construction of the regional maps of honey research shown in Section 3.3.

### 3.2. Global Hotspots of Honey Phenolics Research

**Figure 1:**
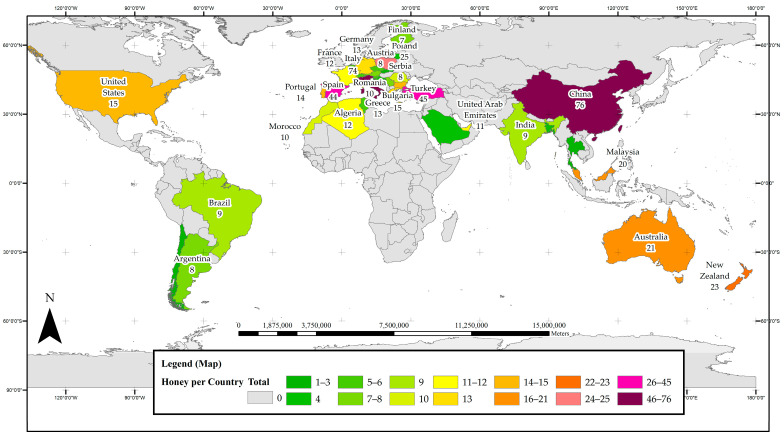
Map of hotspots of honey phenolic research worldwide.

Figure 1 presents the distribution of research on phenolic constituents in honeys across 37 countries. Countries that have yielded a high number of papers on phenolic honey constituents can be considered current ‘hot spots’ for this type of research. China leads the global research efforts with 76 reports on phenolic constituents in monofloral honeys, 76% of which were locally sourced. There were 74 papers originating from Italy with 89% of the reported samples being local honeys, and 45 reports from Turkey, all of which reported on Turkish honeys. Spain was also found to be a hotspot for research on phenolic honey constituents with 44 papers from this country, just under half of them (41%) reporting on locally sourced honeys. A total of 25 studies were carried out in Poland, with the vast majority (92%) investigating Polish honeys, 23 reports came from New Zealand (with 91% of the investigated honeys being local), 21 from Australia and 20 from Malaysia, both of which had 95% of the reported honeys sourced locally. 

### 3.3. Regional Hotspots of Honey Phenolics Research

**Figure 2:**
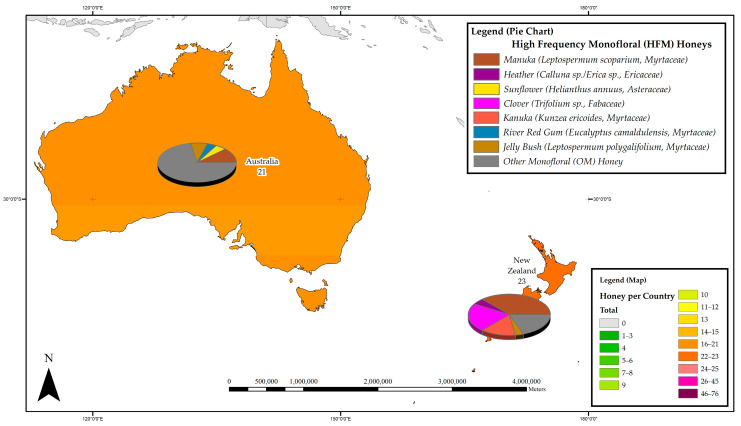
Most frequently studied honeys with respect to their phenolic profile in Australia and New Zealand.

**Figure 3:**
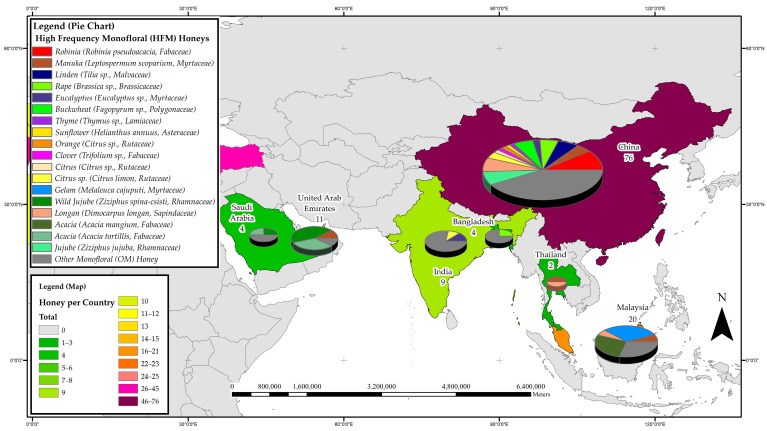
Most frequently studied honeys with respect to their phenolic profile in Asia.

**Figure 4:**
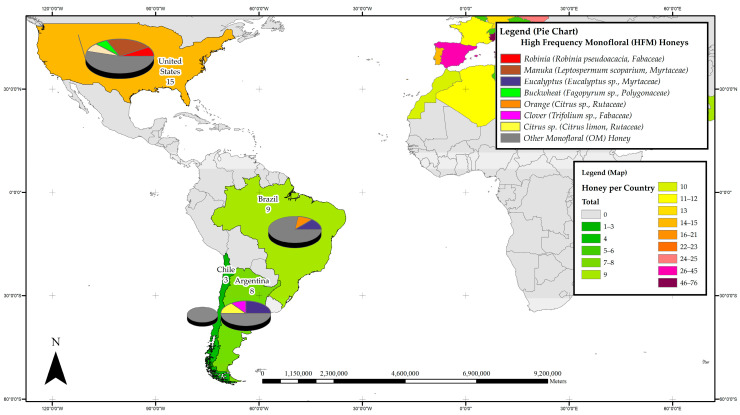
Most frequently studied honeys with respect to their phenolic profile in the Americas.

**Figure 5:**
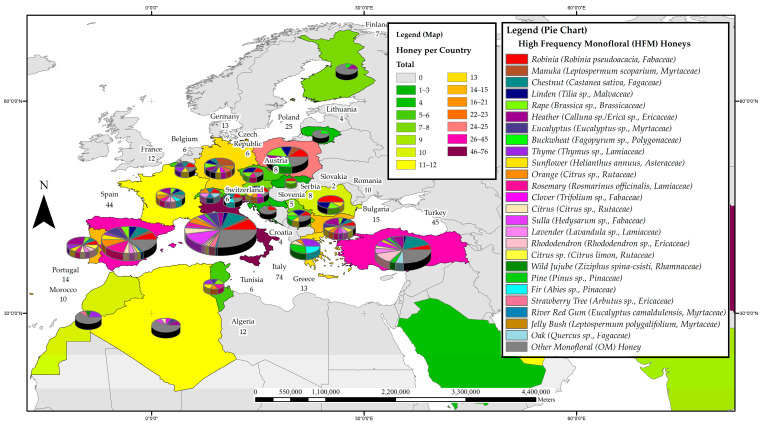
Most frequently studied honeys with respect to their phenolic profile in Africa and Europe.

The distribution of studies on the phenolic constituents of honeys was further divided into four subregions to ascertain the most prevalent monoflorals studied in the respective geographical subregions of Australia and New Zealand; Asia; the Americas, and Africa and Europe. Figure 2, Figure 3, Figure 4 and Figure 5 detail the most frequently studied honeys in the four respective regions, with colour coding used for each region also conveying information on the respective popularity of honey phenolics research. The pie chart included in the maps allows assessing which monofloral honey species these regional research efforts were focused on.

#### 3.3.1. Australia and New Zealand

In Australia and New Zealand, the phenolic constituents of a total of 44 monofloral honeys belonging to 9 plant families have to date been reported, of which 31 belong to the Myrtaceae family, 4 are from the Fabaceae family and 2 from the Proteaceae family. New Zealand leads the region in honey phenolics research with 23 individual reports, 9 of which are attributed to phenolics research on Manuka honey. A total of 21 studies on honey phenolic constituents were carried out in Australia, 10 of which are focused on various Eucalyptus honeys (Myrtaceae). Within the region, Manuka (*Leptospermum scoparium*, Myrtaceae) honeys are the most studied, with 11 reports on their phenolic constituents, followed by Kanuka (*Kunzea ericoides*, Myrtaceae) and Clover honeys (*Trifolium* sp., Fabaceae), each with 4 reports, and Jelly bush honey (*Leptospermum polygalifolium*, Myrtaceae) and Rewarewa honey (*Knightia excelsa*, Proteaceae), with two reports each. Figure 2 details the most frequently studied honeys in the Australia and New Zealand region. 

#### 3.3.2. Asia 

To date, research on honey phenolic constituents has been carried out in seven countries in Asia, namely Bangladesh, China, India, Malaysia, Saudi Arabia, Thailand and the United Arab Emirates. A total of 126 reports have been produced in the region, hotspots of research on honey phenolics being China with 76 studies, Malaysia with 20 and the United Arab Emirates with 11. Considering the botanical origin of the honeys studied in the region, 26 belong to the Fabaceae family, 17 are Myrtaceae, 10 Rhamnaceae and 8 each are Lamiaceae and Sapindaceae. The phenolic constituent profile of Gelam honey (*Melaleuca cajuputi*, Myrtaceae) appears to be the most studied in the region with seven reports, followed by that of New Zealand Manuka honey (*Leptospermum scoparium*), Rape honey (*Brassica* sp., Brasicaceae), Longan honey (*Dimocarpus longan*, Sapindaceae), Robinia honey (*Robinia pseudoacacia* L., Fabaceae) and Wild Jujube honey (*Ziziphus spina-csisti*, Rhamnaceae), which have been addressed in six reports each. Furthermore, the phenolic profile of Acacia honeys from *Acacia mangium* (Fabaceae) and *Acacia tortilis* (Fabaceae), Buckwheat honey (*Fagopyrum esculentum*, Polygonaceae) and Linden honey (*Tilia* sp., Malvaceae) have also been discussed in five studies each. Figure 3 details the most frequently studied honeys in the Asia region with respect to phenolic profile, and it visually conveys the importance of China for honey phenolic research in the region. Not only do the researchers in China report on a relatively large number of monofloral honeys, more than half of the honeys studied can be considered HFMs based on the reports generated. Thus, in many respects, China’s research efforts strongly influence what constitutes, seen through a global lense, a HFM honey.

#### 3.3.3. The Americas

In the Americas region, Argentina, Brazil, Chile and the United States can be considered honey phenolics research hotspots with a total of 35 published studies reporting on the phenolic constituents of monofloral honeys from 16 plant families, 7 each from Fabaceae and Myrtacea. Most studies (15 reports) were carried out in the United States, 9 in Brazil and 8 in Argentina. Interestingly, within the Americas, the New Zealand Manuka honey (*Leptospermum scoparium,* Myrtaceae) was the most studied, with four individual reports, followed by three studies on the phenolic constituents of Eucalypt honey (*Eucalyptus* sp., Myrtaceae) and two each on Azara honey (*Azara* sp., Salicaceae), Schinus honey (*Schinus terebinthifolius*, Anacardiaceae) and Tupelo honey (*Nyssa aquatica*, Nyssaceae). Figure 4 details the most frequently studied honeys in the Americas and it can be seen that, with the exception of the United States, honey phenolic research in this vast region is mainly focused on regionally important honeys classified as OMs in this review, and have, on a global scale, not yet attracted considerable research attention.

#### 3.3.4. Africa and Europe

Research on phenolic constituents in honey has been reported from 24 countries in the Africa and Europe region, totalling 351 reports on monofloral honeys from 31 families. Italy leads the region with 74 reports, followed by Turkey with 45, Spain with 44, and Poland with 25. Honeys of the Fabaceae family were researched the most (53 papers), followed by honeys from the Lamiaceae (47), Ericaceae (39), Myrtaceae (33), Fagaceae (32), Rutaceae (25), Asteraceae (23), Malvaceae (21), Brassicaceae (18) and Pinaceae (13) families. Of these, the Robinia honey (*Robinia pseudoacacia* L., Fabaceae) appears to be the most studied with 29 reports, followed by Chestnut honey (*Castanea sativa* Mill. Fagaceae) with 28, Heather honey (*Calluna* sp./*Erica* sp. Ericaceae) with 23, Linden honey (*Tilia* sp., Malvaceae) with 20, Rape honey (*Brassica* sp., Brasicaceae) with 18, and Eucalypt honey (*Eucalyptus* sp., Myrtaceae) and Thyme honey (*Thymus* sp. Lamiaceae) with 15 reports each. Additionally, attracting attention were the Rosemary honey (*Rosmarinus officinalis* L. Lamiaceae) and Sunflower honey (*Helianthus* sp., Asteraceae) with 13 studies each, as well as Orange honey (*Citrus aurantium/sinensis*, Rutaceae) with 12 and Buckwheat honey (*Fagopyrum esculentum*, Polygonaceae), Sulla honey (*Hedysarum* sp., Fabaceae) and Lavender honey (*Lavandula* sp. Lamiaceae) with 10 reports each. Figure 5 details the most frequently studied honeys in Africa and Europe. From the pie charts, it can be seen that most of the European countries appear to contribute research on HFMs and also tend to have a broader research focus than the African countries. It is also evident that Robinia (*Robinia pseudoacacia* L., Fabaceae) honey seems to attract a lot of research interest across Europe, reflected in the high number of individual research reports on this monofloral honey.

### 3.4. Phenolic Honey Constituents

Table 1 summarises the 170 compounds, 161 of them phenolic in nature, reported from the 159 monofloral honey groups covered by this literature review. Based on existing phenolic compound classifications with minor modifications, the compounds are grouped into five chemical classes, namely simple phenols (two groups), polyphenols, a miscellaneous and an ‘other phenolics’ group as well as non-phenolics [23,24,25,26,27,28]. Simple phenols include phenolic acids, which are chemically defined as carboxylic acid derivatives of phenols and are generally grouped into two subclasses, hydroxylcinnamic derivatives (HCAD group) and hydroxylbenzoic acids derivatives (HBAD group). A total of 20 HCADs and 21 HBADs were reported from honeys around the globe, making them the most common phenolic constituent class in honeys identified to date. Polyphenols, on the other hand, are a group of compounds which are characterized by the existence of more than one phenol unit or building block per molecule and can further be subdivided into two classes, tannins and flavonoids (flavonoid group) with the former being further grouped into hydrolysable and condensed tannins and the latter being divided, for example, into flavones, flavonols, flavanones, dihydroflavonols, chalcones, aurones, isoflavonoids, bioflavonoids [23,26]. 

This review has identified 89 honey constituents from the flavonoid class in total, 12 of them being flavones, 42 flavonols, 11 flavanones, 7 flavanonols, 7 isoflavonoids, 7 flavan-3-ols and 1 each of anthocyanidin, aurone and chalcone. The miscellaneous or ‘other phenolics group’ comprises all other phenolic compounds that do not fall into the above distinct subgroups. Thirty-one honey constituents reported to date belong in this category and include alkylmethoxyphenols (1), alkylphenols (3), hydroxybenzaldehydes (4), hydroxyacetophenones (3), other/miscellaneous phenolics (2), hydroxycoumarins (1), guaiacol (1), anthraquinones (1), naphtoquinones (1), hydroxyphenylacetic acids (6), hydroxyphenyllactic acids (3), hydroxyphenylpropanoic acids (2), hydroxyphenylpentanoic acids (1), benzyl oxalate esters (1), and stilbenes (1) [23,24]. Nine non-phenolic compounds, which were mostly reported as biomarkers for certain honeys, are also included in this review.

Among the reported compounds, caffeic acid (HCAD) is the most prevalent in honeys having been identified in 118 of the 159 investigated monofloral honeys. Gallic acid (HBAD) came in second with 106 reports, followed by p-coumaric acid (HCAD) with 104, ferulic acid (HCAD) with 103 and quercetin (flavonol) with 102 reports. 

When analysing the reported honey constituents along the honeys’ respective botanical classification, it was found that 93, or 55%, of the identified, mostly phenolic compounds in honey have been found in Robinia honey (*Robinia pseudoacacia*, Fabaceae), 76 (45%) in Chestnut honey (*Castanea sativa* Mill., Fagaceae), 75 (44%) in Manuka honey (*Leptospermum scoparium*, Myrtaceae), 69 (41%), respectively, in various Eucalyptus honeys (*Eucalyptus* sp., Myrtaceae), Rape honey (*Brassica* sp. Brassicaceae) and Linden honey (*Tilia* sp., Malvaceae), and 63 (37%) in Sunflower honey (*Helianthus annuus*, Asteraceae).

#### 3.4.1. Flavonoids 

**Figure 6:**
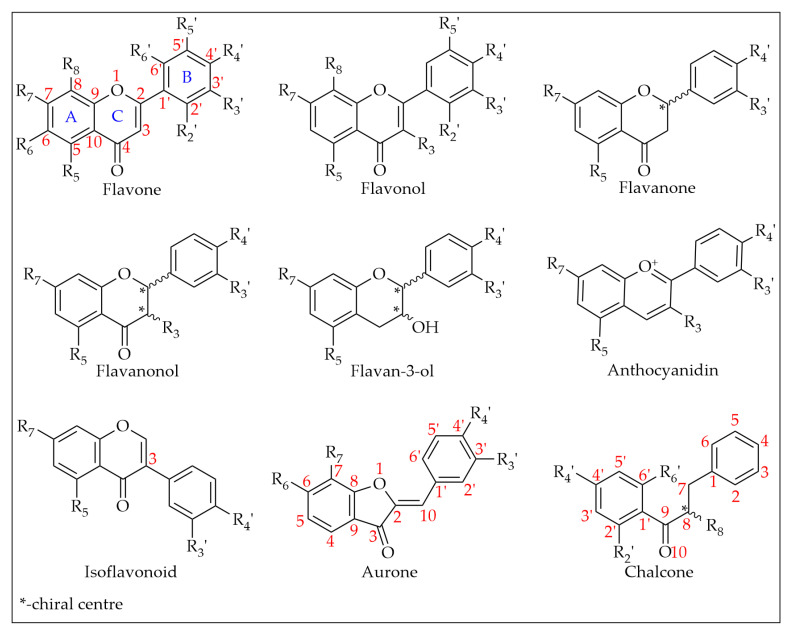
Flavonoid subclasses reported in monofloral honeys.

Chemically, flavonoids can be classified as polyphenols as they possess at least one hydroxyl substituent in their structure. They are made up of a flavane nucleus of 15 carbon atoms (C_6_-C_3_-C_6_) and are diphenyl-propanoids. The C_6_ and C_3_ moieties are arranged to form two fused rings in which the first is an oxygen-containing heterocycle and the second one is a benzene ring constituting a phenylchromane nucleus (2,3-dihydro-2-phenylchroman-4-one). To the base skeleton of the phenylchromane, a second phenyl substituent is linked and, according to the bond position (C2, C3, C4), flavanes, isoflavanes, and neoflavanes, respectively, can be distinguished [26]. These groups usually share a common chalcone precursor and are therefore biogenetically and structurally related [27]. On the other hand, as seen in Figure 6, on the basis of the substitution patterns of the three rings, several subclasses of flavonoids can be identified (e.g., flavones, flavonols, flavanones, flavanonols, isoflavanonoids, flavan-3-ols, and anthocyanidins) [26]. Other natural products such as chalcones and aurones also contain a C_6_–C_3_–C_6_ backbone and are thus considered minor flavonoids [27,28]. Flavonoids may exist as both aglycones and prenylated and methyl ethers, and in glycosylated forms incorporating sugar residues that can be linked to several positions of the three rings in form of both O- and C-glycosides [26].

Seventy percent of the reported flavonoids in honey were found as aglycones, probably due to the action of amylase in bee saliva, which can rapidly cleave glycosidic linkages to liberate flavonoid aglycones from the respective glycosides [29]. 

Flavonoids are synthesised in all parts of a plant and play an important role in providing color, fragrance and taste to fruits, flowers and seeds, making them attractants for insects, birds, and mammals, which aid in pollen and seed transmission [29,159,160,161,162]. However, plants also release numerous chemicals such as flavonoids to deter insects and other predators [159,160]. Aside from that, the strong light absorbance of flavonoids in the ultra-violet region also allows them to act as a protective screen against harmful UV-B radiation [29,162]. They also function as signal molecules, allopathic compounds, phytoalexins, detoxifying agents and antimicrobial defensive compounds [162]. Flavonoids, along with other phenolic compounds are responsible for the organoleptic characteristics of honey [3]. In honey, they originate not only from the nectar but, to an extent, also from plant pollen and plant resins collected by bees. Flavonoids can thus be considered as markers for the botanical and geographical origins of honeys [163] and have associated biological and pharmacological activities such as antioxidant [27,162,164,165], antimicrobial [164], anticancer [164,166,167], anti-inflammatory [162,164], antiallergic [164], antithrombotic [164], cardioprotective [164], hepatoprotective [164,168] neuroprotective [164], antimalarial [161], antileishmanial [161], anticholinesterase [162], anti-Alzheimer’s disease [169], antiulcer [164], antiatherosclerotic [164], antidiabetic [164], estrogenic effect [27], steroid-genesis modulators [162], vasorelaxant effect [164], improved blood flow [170], the inhibition of cholesterol absorption [171], countering antibiotic resistance [162], and protection from damage by ultraviolet B radiation [172]. 

Based on the findings of this study, the vast majority of monofloral honeys included in this review (82%) were reported to contain flavonoids, 89 different types in total. Robinia honey (*Robinia pseudoacacia*, Fabaceae) was found to contain 53 of the identified flavonoids in honey. 35 flavonoids, respectively, have to date been identified in Eucalyptus honey (*Eucalyptus* sp., Myrtaceae) and Linden honey (*Tilia* sp., Malvaceae), 34 in Chestnut honey (*Castanea sativa* Mill., Fagaceae) and 32 each in Manuka (*Leptospermum scoparium*, Myrtaceae) and Rape (*Brassica* sp. Brassicaceae) honeys.

##### Flavones

**Figure 7:**
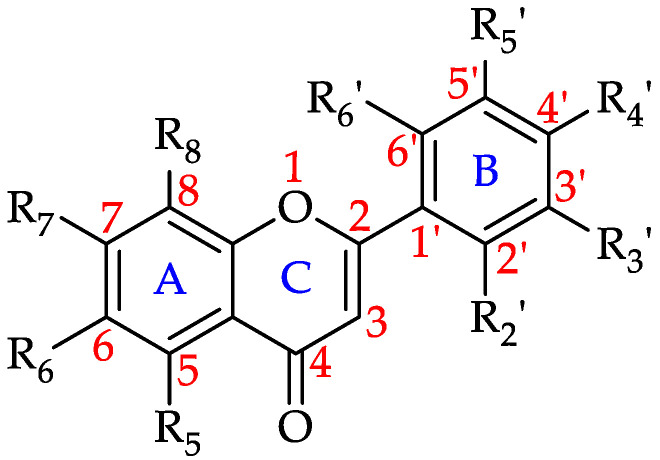
Basic flavone structure (see **1**–**12** in Table 2).

Flavones are a subclass of flavonoids that contain a double bond between C2 and C3 in the flavonoid skeleton, no substituent on the C3 position and the C4 position is oxidised (Figure 7). Along with flavonols, flavones are the primary pigments in white- and cream-colored flowers and act as copigments with anthocyanins in blue flowers. They also act as UV-B protectants in plants as they absorb in the 280–315 nm range [173]. 

Based on the findings of this comprehensive review, at least one flavone has to date been reported to be present in 64% of the monofloral honeys, with Robinia honey (*Robinia pseudoacacia*, Fabaceae) containing nine different flavones. Chrysin has been found to be the most common flavone, reported to be present in 83 monofloral honeys, followed by apigenin in 74, luteolin in 69, tectochrysin in 16 and acacetin in 15 honeys. Table 2 shows all the flavones that have to date been identified in monofloral honeys and the number of honeys in which they were identified. 

#### Flavonols

**Figure 8:**
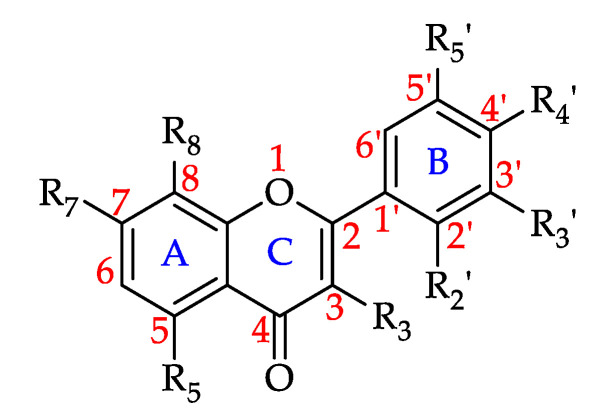
Basic flavonol structure (see **13**–**54** in Table 3).

Flavonols are naturally yellow in color (*flavus* is Latin for yellow) and are present in plant and fungi [174]. They are also known as 3-hydroxyflavones, the only difference to flavones being the hydroxyl group at C3 position. Flavonols are frequently found as *O*-glycosides, with glycosidation occuring mainly at the 3-position of the C-ring (Figure 8) [175]. Flavonols are primarily accrued in the epidermal cells of plant tissues and serve as a protection against solar radiation, especially UV-B. They also play an important role, along with xanthophylls, in protecting the photosynthetic apparatus in situ from excess solar radiation and are known to moderate drought-related oxidative damage because of their strong radical scavenging activity [176].

Based on the findings of this review, more than 74% of the honeys were found to contain at least one of the 42 reported flavonols. Acacia honey (*Robinia pseudoacacia*, Fabaceae) has the highest number of published studies reporting on its flavonols, followed by Eucalyptus honey (*Eucalyptus* sp., Myrtaceae) with 17 studies. Quercetin is the most commonly isolated flavonol reported to be present in 102 monofloral honey groups, followed by kaempferol in 89 honeys, galangin in 66, rutin in 58, myricetin in 54, and isorhamnetin in 43 monofloral honeys. Table 3 shows all the flavonols that have to date been identified in monofloral honeys and the number of monofloral honey groups for which they were reported.

#### Flavanones

**Figure 9:**
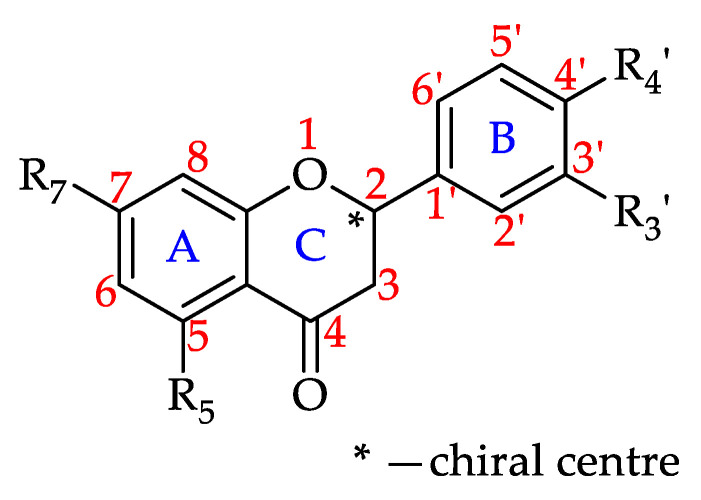
Basic flavanone structure (see **55**–**65** in Table 4).

Flavanones, also referred to as dihydroxyflavones, are characterised by the lack of a double bond between C2 and C3 in the C-ring of the flavonoid skeleton, resulting in a chiral center at C2 (Figure 9) [177]. The chirality creates an angle between the B-ring relative to the A–C rings. This variation in the molecule’s structural orientation impacts flavanones’ interactions with biological receptors, in turn influencing their bioactivities [178,179].

Based on the findings of this review, 62% of the honeys were reported to contain at least one of the 11 flavanones that to date have been isolated from honeys. Robinia honey (*Robinia pseudoacacia*, Fabaceae) has been found to contain nine of these flavanones. Pinocembrin has been identified in 64 monofloral honeys, followed by naringenin found in 54 and hesperitin in 49 honeys, respectively. Table 4 shows all the flavanones that have to date been identified in monofloral honeys and the number of honeys they have been reported to be present in.

#### Flavanonols

**Figure 10:**
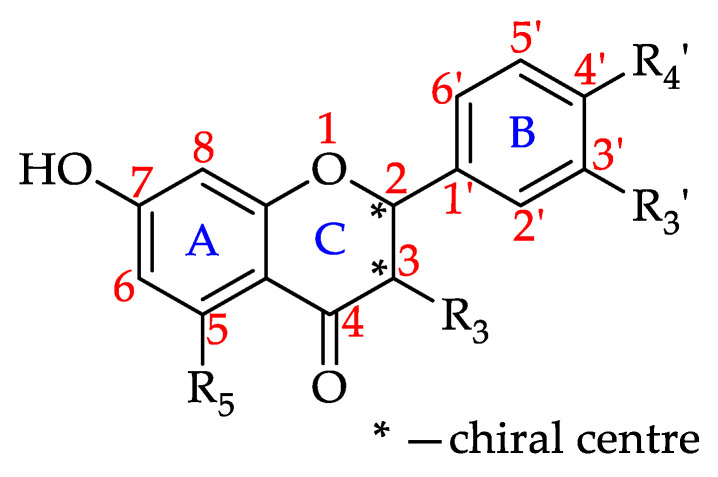
Basic flavononol structure (see **66**–**72** in Table 5).

Flavanonols, which are also known as dihydroflavonols, are 3-hydroxy derivatives of flavanones (Figure 10) [180]. This review found that the presence of at least one of the seven flavanonols that have to date been isolated in honey, was reported for 32% of the monofloral honeys. Four of these seven flavanonols were identified in Robinia honey (*Robinia pseudoacacia*, Fabaceae). Pinobanksin is the most prevalent flavanonol, reported to be present in 49 honeys. Table 5 shows all the flavanonols that that have to date been identified in monofloral honeys and the number of monofloral honeys in which they were found.

#### Flavan-3-ols

**Figure 11:**
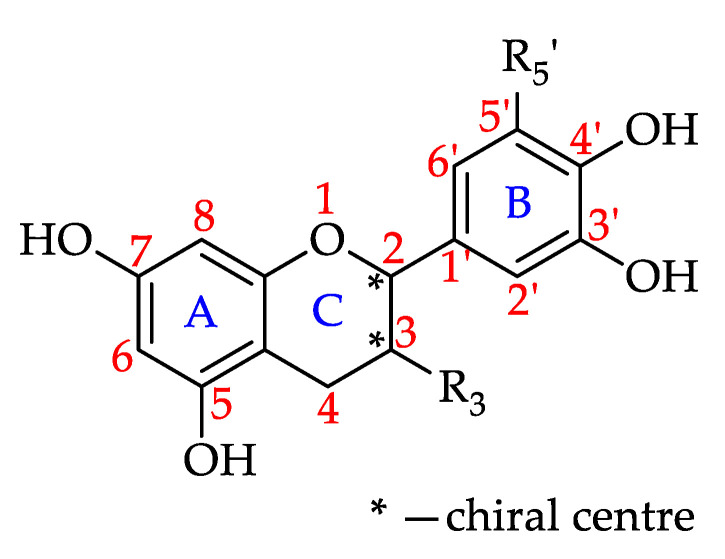
Basic flavan-3-ol structure (see **73**–**80** in Table 6).

Flavan-3-ols or flavanols are also known as catechins. They are characterised by the absence of a double bond between C2 and C3 as well as the absence of a carbonyl on C4 of ring C. As a result, flavan-3-ols feature two chiral carbons and can form four possible diastereomers [181,182]. They exist in both monomeric (catechins) and in polymeric (proanthocyanidins) forms. The monomeric form can vary in its degree of hydroxylation at position 5 and 7 on ring A and at positions 3′, 4′ and 5′ on ring B. C3 of ring C usually carries a hydroxyl group or is esterified with gallic acid (gallate) (Figure 11) [183]. The polymeric form, also known as condensed tannin, features dimers, trimers, oligomers and polymers of flavan-3-ol units linked by C–C bonds either at 4–6 (A-type proanthocyanidins) or 4–8 (B-type proanthocyanidins). They are also classified as procyanidins when derived from catechin, epicatechin and their gallic esters [183].

Seven flavan-3-ols have to date been identified in honeys with at least one flavan-3-ol reported to be present in just over a third (34.6%) of the monofloral honey groups. Five different flavan-3-ols have been identified in Sage honey (*Salvia officinalis* L., Lamiaceae), making it the honey with the highest number of reported flavan-3-ols. Catechin and epicatechin are the most prevalent flavan-3-ols in honeys, being present in 29 and 24 honeys, respectively. Table 6 shows all the flavan-3-ols that have to date been identified in monofloral honeys and the number of honeys for which their presence has been reported.

#### Isoflavonoids

**Figure 12:**
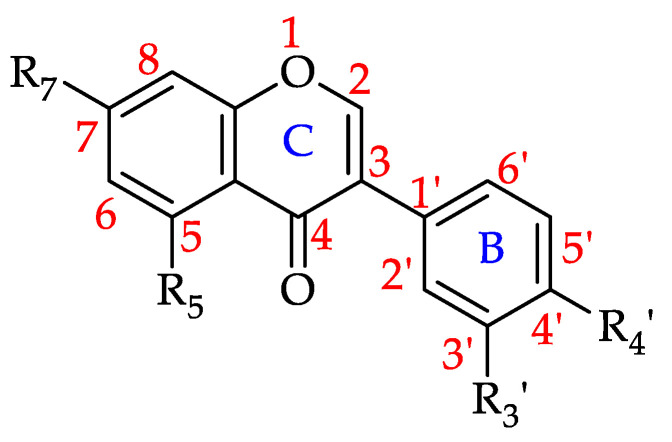
Basic isoflavonoid structure (see **81**–**87** in Table 7).

The structure of isoflavonoids is somewhat different to that of other flavonoids in so far that ring B is connected to C3 of ring C instead of C2 (Figure 12) [184]. Seven isoflavonoids have to date been identified in honey. They do not appear to be a particularly common honey constituent class as only 17% of the monofloral honeys covered by this review were found to contain them. Amonst them, Robinia honey (*Robinia pseudoacacia*, Fabaceae) was reported to contain six different isoflavonoids. Genistein is the most common identified isoflavonoid in honeys with 23 reports, followed by formononetin with 8 reports. Table 7 shows all the isoflavonoids that have to date been identified in monofloral honeys and the number of honeys they have been found in.

#### Aurones and Chalcones

**Figure 13:**
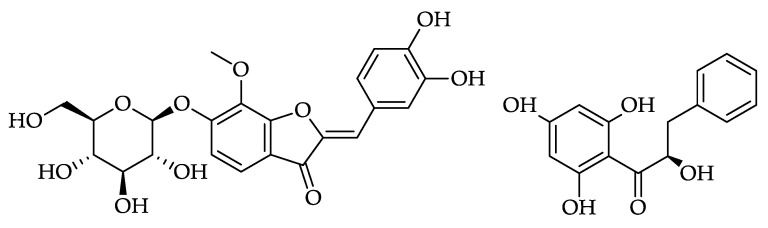
Structure of leptosin (**88**, Table 8) and of pinobanksin chalcone (**89**, Table 8).

Due to their bright yellow color, the word aurones is derived from the Latin word *aurum* for gold. Aurones are considered a minor class of flavonoids. They also contain 15 carbon atoms, arranged in the general structure C_6_–C_3_–C_6_ (Figure 13). They occur in hydroxylated, methoxylated or glycosylated forms [185]. The word chalcone, on the other hand, is derived from the Greek word chalcos, meaning bronze, reflecting the typical colour of most natural chalcones [186]. Chalcones are α,β-unsaturated ketones (*trans*-1,3-diaryl-2-propen-1-ones) consisting of two aromatic rings attached to an α,β-unsaturated carbonyl system with a variety of substituents (Figure 8) [187]. Aurones and chalcones were only identified in 3% and 4%, respectively, of the monfloral honeys covered by this review. Table 8 details these compounds and the number of monofloral honey groups that were found to contain them. 

#### 3.4.2. Hydroxycinnamic Acid and Its Derivatives 

**Figure 14:**
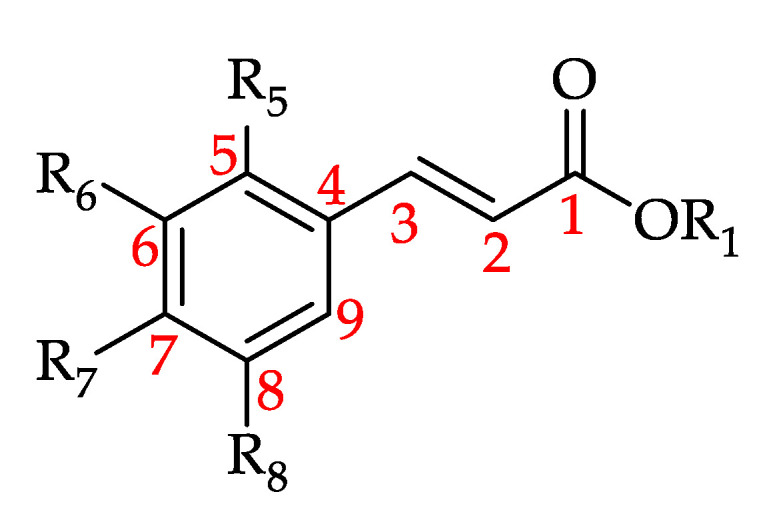
Basic structure of hydroxycinnamic acid and its derivatives (HCADs) (see **90**–**109** in Table 9).

Hydroxycinnamic acid and its derivatives (HCADs) are phenolic acids that are prevalent in plants [188]. They can be considered hydroxy metabolites of cinnamic acid featuring a C6–C3 backbone (Figure 14) [189,190].

A high proportion, 88%, of the monfloral honey groups covered by this review were reported to contain at least 1 of the 20 HCADs that have to date been identified in honeys. Robinia honey (*Robinia pseudoacacia*, Fabaceae) had the highest number of HCADs, 15 in total, while 12 HCADs each were reported for Rape (*Brassica* sp., Brassicaceae) and Sunflower (*Helianthus annuus*, Asteraceae) honeys. Among the HCADs, caffeic acid appears to be the most prevalent, having been reported in 117 of the honeys, followed by p-coumaric acid in 103, ferulic acid in 102, chlorogenic acid in 85 and t-cinnamic acid in 57 honeys. Table 9 shows all the HCADs that have to date been identified in monofloral honeys and the number of honeys in which they were found to be present.

#### 3.4.3. Hydroxybenzoic Acid and Its Derivatives

**Figure 15:**
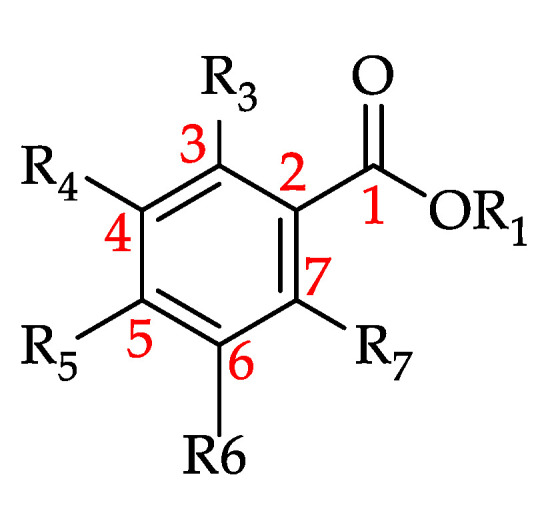
Basic structure of hydroxybenzoic acid and its derivatives (HBADs) (see **110**–**130** in Table 10).

**Figure 16:**
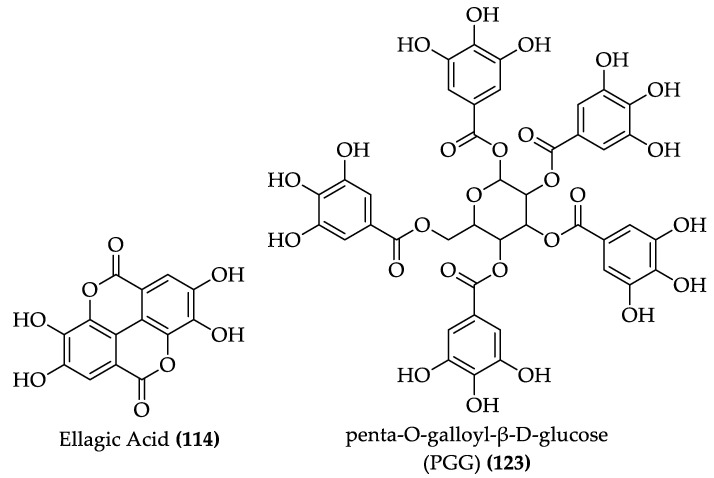
Structure of ellagic acid and penta-O-galloyl-β-D-glucose (PGG) (**122–123**).

Hydroxybenzoic acid and its derivatives (HBADs) are phenolic metabolites featuring the general structure C6 ± C1 (Figure 15 and Figure 16) [191,192]. Of the monofloral honey groups covered by this review, 90% have been reported to contain at least one of the 21 HBADs that have to date been identified in honeys. Chestnut honey (*Castanea sativa* Mill., Fagaceae) and Manuka honey (*Leptospermum scoparium*, Myrtaceae) are reported to contain 16 of the HBADs, while Rape honey (*Brassica* sp., Brassicaceae) and Clover honey (*Trifolium* sp., Fabaceae) contain 15 each. Gallic Acid is the most prevalent HBAD with 105 reports, followed by syringic acid with 85, p-hydroxybenzoic acid with 79, vanillic acid with 66 and protocatechuic acid with 57. Table 10 shows all the HBADs that have to date been identified in monofloral honeys and the number of honeys which were found to contain them.

#### 3.4.4. Miscellaneous and ‘Other’ Phenolics

**Figure 17:**
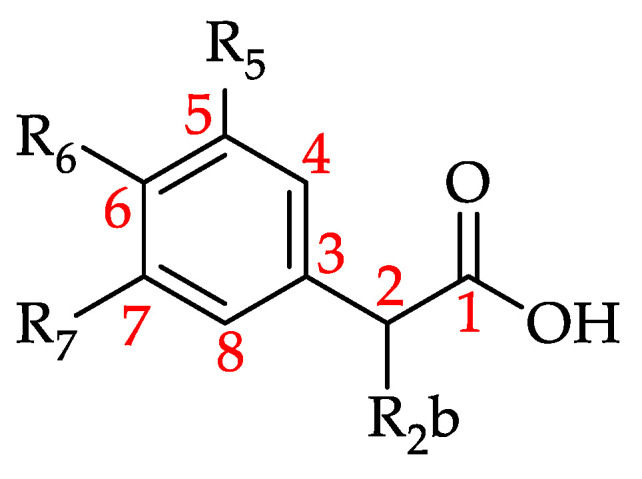
Basic structure of hydroxyphenylacetic acid and derivatives (HPAAD) (see **131–136** in Table 11).

**Figure 18:**
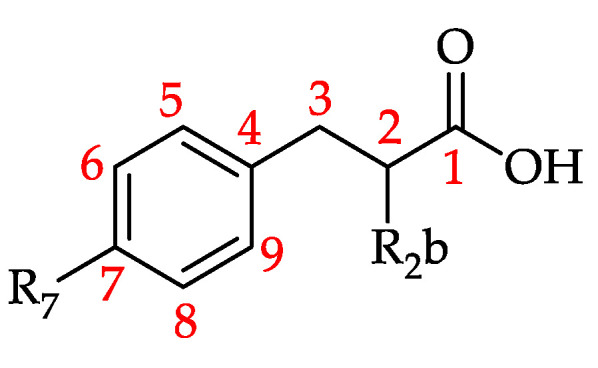
Basic structure of hydroxyphenyllactic acid and derivatives (HPLAD) (see **137–139** in Table 12) and hydroxyphenylpropanoic acid and derivatives (HPPAD) (see **140** and **141** in Table 12).

**Figure 19:**
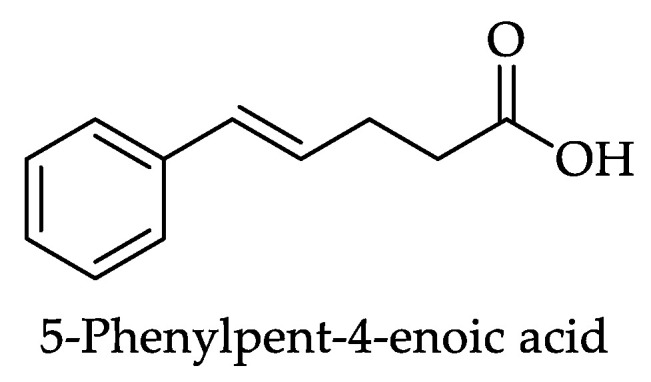
Structure of 5-phenylpent-4-enoic acid, a hydroxyphenylpentanoic acid (HPPeA) (**142**, Table 13).

**Figure 20:**
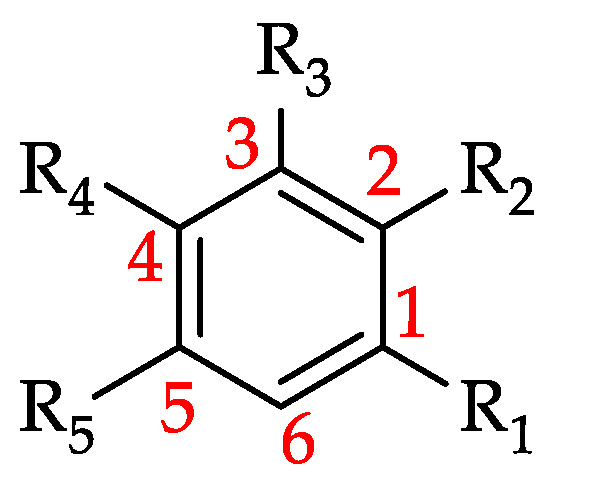
Basic structure of other phenolic compounds (see **143–156** in Table 14).

**Figure 21:**
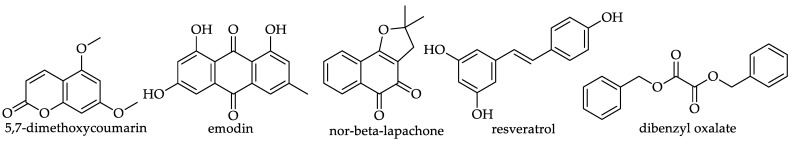
Structure of 5,7-dimethoxycoumarin (**157**, Table 15), emodin (**158**, Table 15), nor-β-lapachone (**159**, Table 15), resveratrol, a stilbene (**160,** Table 15), and dibenzyl oxalate, an oxalate ester (**161,** Table 15) reported in honeys.

Some uncommon phenolic compounds were also identified in the monofloral honeys. This miscellaneous or ‘other’ phenolics group comprises 31 phenolic compounds that do not fall into the subgroups discussed previously. They include six hydroxyphenylacetic acids (HPAAD) (Figure 17 and Table 11); three hydroxyphenyllactic acids (HPLAD) and two hydroxyphenylpropanoic acids (HPPAAD) (Figure 18 and Table 12); one hydroxyphenylpentanoic acid (Figure 19 and Table 13); one alkylmethoxyphenol, three alkylphenols, four hydroxybenzaldehydes and three hydroxyacethophenones, one guaiacol, and two other/miscellaneous phenolic compounds (Figure 20 and Table 14), one hydroxycoumarin, anthraquinone, naphtoquinone, benzyl oxalate ester, and stilbene, respectively (Figure 21 and Table 15). 

Slightly over one-quarter (27%) of the monofloral honey groups covered by this review were reported to contain one or more of the miscellaneous or ‘other’ phenolic compounds. A total of 20 of them were identified in Chestnut honey (*Castanea sativa* Mill., Fagaceae), 19 in Robinia honey (*Robinia pseudoacacia*, Fabaceae), 17 in Lavender honey (*Lavandula* sp., Lamiaceae) and 15 in Clover honey (*Trifolium* sp., Fabaceae). Protocatechualdehyde (hydroxybenzaldehyde) was reported in 40 studies, homogentisic acid (hydroxyphenylacetic acid-HPAAD) in 24, DL-β-phenyllactic acid (hydroxyphenyllactic acid HPLAD) in 23, vanillin (hydroxybenzaldehyde) in 18, phenylacetic acid (HPAAD) and p-hydroxyphenylacetic acid (HPAAD) in 17 each and 3-phenyl propionic acid (hydroxyphenylpropanoic acids-HPPAD) was identified in 13 reports on honey constituents. Table 11, Table 12, Table 13, Table 14 and Table 15 show all the miscellaneous/‘other’ phenolic constituents that that have to date been identified in monofloral honeys and the number of honeys for which their presence has been reported.

#### 3.4.5. Non-Phenolic Compounds

**Figure 22:**
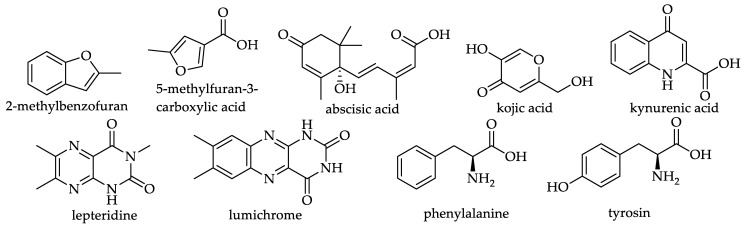
Structures of non-phenolic compounds (see **162–170** in Table 16).

Nine non-phenolic compounds were also reported in 26.7% of the monofloral honey groups covered by this review. Manuka honey (*Leptospermum scoparium*, Myrtaceae) was reported to contain 6 of the 9 non-phenolic compounds, 5 were identified in Kanuka honey (*Kunzea ericoides*, Myrtaceae) and 4 in Eucalyptus honey (*Eucalyptus* sp., Myrtaceae). Absiscic acid, which has been detected in 36 honeys, is the most commonly reported non-phenolic honey constituent. Figure 22 and Table 16 detail the different non-phenolic compounds identified to date in the monofloral honeys.

### 3.5. Analytical Methods Used in Compound Detection

Table 17 details the different analytical methods found in this review for the detection of phenolic compounds in the monofloral honeys. It is evident that the phenolic compounds were mostly identified by high-performance liquid chromatography (HPLC) (67%) using either UV, UV–Vis, UV–UV, photodiode array (DAD or PDA), DAD–UV, electron capture (ECD), or EDC–UV as detectors. Almost one-quarter (24%) of the reports indicated the use of liquid chromatography coupled with mass spectrometry (LC–MS), 5% used a combination of HPLC, LC–MS and/or gas chromatography coupled with mass spectrometry (GC–MS), 1% of the analyses used gas chromatography coupled with mass spectrometry (GC–MS) and high-performance thin-layer chromatography (HPTLC), respectively, and finally, less than 1% used fluorescence spectroscopy to identify the phenolic compounds.

## 4. Conclusions

This review investigated 130 original research articles that detailed the phenolic compounds identified in 556 monofloral honeys. The honeys from 51 botanical families were grouped into 159 monofloral groups. Most of the monofloral honeys belonged to the Myrtaceae and Fabaceae families. The Robinia honey (*Robinia pseudoacacia*, Fabaceae), Manuka honey (*Leptospermum scoparium*, Myrtaceae) and Chestnut honey (*Castanea* sp., Fagaceae) were the most studied monofloral honeys for their phenolic constituents. China, Italy and Turkey were the major hubs the honey phenolic research. A total of 161 phenolic compounds were reported in the honeys and these were classified in this review into five major compound groups, namely flavonoids, hydroxycinnamic acid and its derivatives (HCAD), hydroxybenzoic acid and its derivatives (HBAD), miscellaneous or ‘other phenolics’, as well as nine non-phenolics which were mainly used as marker compounds for specific monofloral honeys. Hydroxycinnamic acid derivatives (HCAD) and hydroxybenzoic acid derivatives (HBAD) were the most prevalent phenolic constituents in the monofloral honeys, with caffeic acid, gallic acid, ferulic acid, and quercetin being the most reported phenolic compounds. Robinia honey (*Robinia pseudoacacia*, Fabaceae), Chestnut honey (*Castanea sativa* Mill., Fagaceae), and Manuka honey (*Leptospermum scoparium*, Myrtaceae) were the monofloral honeys for which the highest number of phenolic compounds has to date been identified. Most of these phenolic compounds were detected and structurally identified using HPLC.

The information compiled in this review can serve as a guide for future research into the identification of phenolic compounds in honey. It illustrates which geographical locations are very active in phenolics research in honey. It also provides information for which monofloral honeys worldwide phenolic compounds have already been determined. Moreover, it also details the specific phenolic constituents that have to date been detected in monofloral honeys and the analytical methods used to identify them. In doing so, it assists with the identification of common or ubiquitous phenolic honey constituents and those that to date have only been found in specific monofloral honeys or honeys derived from particular botanical families.

## Figures and Tables

**Table 1:** Summary of reported phenolic and other compounds in different monofloral honeys.

No	Plant Family	Scientific Name/s	Common Name/s	Country (Research Location)	No. of Study/s	Flavonoids	HCAD	HBAD	Misc./Other Phenolics	NP	Total	Ref
1.	Acanthaceae	*Avicennia germinans Jacq.*	Black Mangrove	Italy	1	Isor, Kaem, Kaem-8-ME, Quer, Quer rham,	CA, p-CouA	SyrA, VA	N.I.	N.I.	9	[30]
2.	Anacardiaceae	*Schinus terebinthifolius*	Mastic, Hawaiian Christmas berry	Brazil, USA	2	Quer	t-CA	GA	N.I.	N.I.	3	[31,32]
3.	Annarrhinum sp.	*Annarrhinum* sp.	Annarrhinum	Algeria	1	Api, Chr, Lut, Gal, Isor, Kaem, Kaemf, Quer, Pinoc, Pinob, Dai, Gene	CA, p-CouA	BenA, ProA, p-HBA, SyrA, VA	N.I.	N.I.	19	[33]
4.	Apiaceae	*Ammi visnaga* L.	Bochnikha	Morocco	1	N.I.	CA, FA, p-CouA, RosA	GA, SyrA	N.I.	N.I.	6	[34]
5.	Apiaceae	*Apiaceae* sp.	Apiaceae	Algeria	1	Api, Chr, Lut, Gal, Isor, Kaem, Quer, Quer rham, Hest, Isosak, Pinoc, Pinob, Gene	CA, FA, p-CouA, t-CA	BenA, ProA, p-HBA, VA, SyrA	3,4-DHPAA, p-HPAA	N.I.	24	[33]
6.	Apiaceae	*Daucus* sp.	Wild Carrot	Belgium	1	Chr, Pinoc	CA, p-CouA	N.I.	N.I.	N.I.	4	[35]
7.	Apiaceae	*Eryngium campestre* L.	Common Eryngo	Turkey	1	N.I.	CA, FA, p-CouA,	p-HBA	N.I.	N.I.	4	[36]
8.	Apiaceae	*Foeniculum vulgare*	Fennel	China	1	Chr, Lut, Vit, Fis, Gal, Isor, Kaem, Quer, Hest, Hesd, Nar, Pinoc, Sak, Pinob, Tax, Form,	CA, ChloA, CChloA, FA, IfA, p-CouA, SinA,	GA, ProA, p-HBA, SalA, SyrA	N.I.	N.I.	28	[37]
9.	Aquifoliaceae	*Ilex* sp.	Gallberry	USA	1	Chr, Gal Kaem, Quer, Rut, Hest, Pinoc	p-CouA	N.I.	N.I.	N.I.	8	[38]
10.	Arecaceae	*Cocos nucifera*	Coconut	Malaysia	1	N.I.	CA	BenA, GA	N.I.	N.I.	3	[39]
11.	Arecaceae	*Cynara cardunculus*	Cardoon	Italy	1	Api, Gal Quer Pinob, Pinoc	N.I.	SyrA	N.I.	N.I.	6	[40]
12.	Arecaceae	*Serenoa repens*	Palmetto	USA	1	Chr, Lut, Gal, Kaem, Quer, Rut, Hest, Pinoc	p-CouA	N.I.	N.I.	N.I.	9	[38]
13.	Asphodelaceae	*Aloe vera barbadensis*	Aloe	Saudi Arabia	1	Chr, Lut, Gal, Kaem Myr, Quer, Nar	CA, ChloA, p-CouA	GA, p-HBA, SyrA	p-HPAA	N.I.	14	[41]
14.	Asphodelaceae	*Asphodelus* sp., *A. microcarpus Salzm. and Viv.*	Asphodel, Asphodelus	Italy	2	Api, Gal, Quer, Pinoc, Pinob	FA	MS, SyrA,	N.I.	PhAn, Tyr	10	[40,42]
15.	Asteraceae	*Cardus* sp.	Thistle	Italy	1	N.I.	N.I.	N.I.	DL-β-PLA	Lum, PhAn, Tyr	4	[42]
16.	Asteraceae	*Centaurea dumulosa*	Morar	Morocco	1	N.I.	CA, FA, p-CouA, RosA	GA, SyrA	N.I.	N.I.	6	[34]
17.	Asteraceae	*Chrysanthemum* sp.	Chrysanthemum	China	1	N.I.	CA	GA, p-HBA, SyrA	Prod	N.I.	5	[43]
18.	Asteraceae	*Cirsium discolor*	Cardo	Italy	1	Api, Chr, Lut, Gal, Kaem, Myr, Quer, Pinoc, Pinob	CA, FA, p-CouA,	SyrA, VA	N.I.	N.I.	14	[44]
19.	Asteraceae	*Conyza bonariensis*	Rabat	Morocco	1	N.I.	CA, FA	SyrA	N.I.	N.I.	3	[34]
20.	Asteraceae	*Echinops spinosissimus*	Morar Akhdar	Morocco	1	N.I.	CA, RosA	N.I.	N.I.	N.I.	2	[34]
21.	Asteraceae	*Gochnatia* sp.	Cambara	Brazil	1	Chr, Gal, Nar	t-CA, m-MCA, m-CouA	BenA, GA, SyrA	N.I.	AbsA	10	[45]
22.	Asteraceae	*Helianthus annuus* L.	Sunflower	Australia, Austria, Belgium, Bulgaria, China, France, Germany, Italy, Portugal, Romania, Serbia, Spain, Tunisia, Turkey	15	Aca, Api, Chr, Lut, Tec, Gal, Gal-3-ME, Isor, Kaem, Kaem-8-ME, Kaem-3-O-(6”-acetyl)-β-Gluc, Mor, Myr, Myr-3,7,4′5′-TeME, Quer, Quer-3,3-DME, Quer-3,7-DME, Quer-3-ME, Rut, Hest, Nar, Pinoc, Pinos, Pinob, Pinob-3-O-ace, CG	CA, CADAE, CAPE, ChloA, FA, m-CouA, MF, o-CouA, p-CouA, SinA, t-CA, t-p-CouAME,	BenA, ElA, GA, GenA, M-4-HBz, MS, ProA, p-HBA, ResA, SalA, SyrA, VA, VAME,	3,4-DHPAA, HGA, MandA, PAA, p-HPAA, DL-β-PLA, 3-PPA, PhlA, 4-MPC, Prod, Van,	AbsA	63	[35,46,47,48,49,50,51,52,53,54,55,56,57,58,59]
23.	Asteraceae	*Pluchea Sagittalis*	Quitoco	Brazil	1	Quer	N.I.	GA	N.I.	N.I.	2	[31]
24.	Asteraceae	*Solidago virgaurea* L.	Goldenrod	China, Poland, Serbia	3	Api, Chr, Lut, Gal, Kaem, Myr, Quer, Rut, Hest, Nar, Pinoc, EC, Gene	CA, ChloA, FA, p-CouA, t-CA	2,3,4-THBA, ElA, GA, GenA, ProA, p-HBA, VA	Prod	N.I.	26	[43,54,60]
25.	Asteraceae	*Taraxacum officinalis*	Taraxacum, Dandelion	Austria, Italy, Spain	3	Aca, Api, Chr, Lut, Tec, Gal, Isor, Kaem, Kaem-8-ME, Myr, Quer, Quer-3-ME, Isosak, Pinoc, Pinob	CAPE, FA, MF, p-CouA	MS, VAME	N.I	N.I	21	[51,57,61]^,^
26.	Asteraceae	*Vernonia* sp.	Assa peixe	Brazil	1	Chr	ChloA, FA, t-CA	BenA, GA, PAA, ProA	N.I	AbsA	9	[45]
27.	Boraginaceae	*Borago officinalis*	Blue borage	China	1	Api, Chr, Tang, Hest, Nar	CA, ChloA	2,3,4-THBA, GA, p-HBA, SyrA	Prod	N.I.	12	[43]
28.	Boraginaceae	*Echium plantagineum*	Echium	Bulgaria	1	N.I.	CA, FA, o-CouA, p-CouA, t-CA	BenA, ProA	PAA, DL-p-HPLA, DL-β-PLA,	N.I.	10	[59]
29.	Brassicaceae	*Brassica* sp., *B. campestri,* *B. campestris* L., *B. napus,* *B. napus* L., *B. napus oleifera*, *B. nigra,* *B. rapa*, *B. napus* L. *var. oleifera Metzger*	Oilseed, Rape, Rapeseed, Canola, Mustard flower	Austria, Bangladesh, Bulgaria, China, Czech Republic, France, Germany, Poland, Portugal, Romania, Serbia, Slovakia, Spain, Tunisia	24	Aca, Api, Bai, Chr, Lut, Tec, Vit, Gal Gal-3-ME, Isor, Kaem, Kaem-8-ME, Kaem-3-O-(6”-acetyl)-β-Gluc, Mor, Myr, Myr-3,7,4′5′-TeME, Quer, Quer-3,7-DME, Quer-3-ME, Querc, Rham, Rut, Erio, Hest, Hesd, Isosak, Nar, Pinoc, Pinos, Pinob, Pinob-3-O-ace, Ono	CA, CADAE, CAPE, ChloA, FA, IfA, o-CouA, p-CouA, p-MCA, RosA, SinA, t-CA	BenA, ElA, GA, GenA, M-4-HBz, MS, m-HBA, PAA, ProA, p-HBA, ResA, SalA, SyrA, VA, VAME	3,4-DHPAA, HGA, MandA, PAA, p-HPAA, DL-β-PLA, 3-PPA, PhlA, 4-MPC	AbsA	69	[46,47,48,49,51,52,54,55,57,59,62,63,64,65,66,67,68,69,70,71,72,73]
30.	Brassicaceae	*Diplotaxis tenuifolia*	Diplotaxis	Argentina	1	Chr, Tec, Isor, Isor-4′-diGlc, Isor-4′-gent, isor-4′-Glc, Isor-3-Glc-4′-gent, Kaem, Kaem-3-diGlc isomer, Kaem-3-soph, Kaem-4′-Glc, Quer, Quer-3,3′,4′-triGlc, Quer-3,4′-diGlc, Quer-3-soph, Pinoc, Pinos, Pinob	CADAE, ChloA, FA, p-CouA	N.I.	N.I.	N.I.	22	[74]
31.	Bromeliaceae	*Ananas comosus*	Nenas, Pineapple	Malaysia	3	Chr, Kaem, Myr, Quer, Rut, Hest, Nar	CA, ChloA, FA, p-CouA	BenA, GA, SyrA, ElA	N.I.	N.I.	15	[75,76,77]
32.	Cactaceae	*Opuntia*	Prickly pear	Italy	1	Chr, Kaem, Myr, Quer, Rut, Pinoc, EC	FA, SinA	GA	N.I.	N.I.	10	[78]
33.	Campanulaceae	*Codonopsis pilosula (Franch.) Nannf.*	Codonopsis	China	2	Api, Bai, Chr, Lut, Vit, Kaem, Myr, Quer, Querc, Rut, Hest, Hesd, Nar, Pinoc, EGC, Cal, Form, Gene, Ono, Cal-7-O-β-D-gluc	CA, CAPE, ChloA, FA, IfA, p-CouA, SinA	BenA, GA, m-HBA, ProA, p-HBA, SalA, SyrA	N.I.	AbsA	35	[55,62]
34.	Capparaceae	*Capparis* sp., *Capparis spinosa*	Capparis, Caper, Kabbar	Algeria, Morocco	2	Api, Chr, Lut, Gal, Isor, Kaem, Kaemf, Myr, Quer, Quer-rham, Pinoc, Pinob, EC, Dai, Gene	CA, FA, p-CouA, RosA, t-CA	BenA, ProA, GA, p-HBA, SyrA, VA	3,4-DHPAA, HVA, p-HPAA	N.I.	29	[33,34]
35.	Caprifoliaceae	*Lonicera* sp.	Honeysuckle	China	1	Lut	N.I.	N.I.	N.I.	N.I.	1	[43]
36.	Cistaceae	*Cistus* L.	Cistus	Italy	1	Api, Lut, Gal, Kaem, Pinoc, Pinob	t-CA	SyrA	N.I.	N.I.	8	[40]
37.	Convolvulaceae	*Ipomoea triloba* L.	Morning Glory	Italy	1	Isor, Kaem, Kaem-8-ME, Kaem-7-O-rham, Quer	CA, FA, p-CouA,	SyrA, VA	N.I.	N.I.	10	[30]
38.	Convolvulaceae	*Turbina corymbosa* (L.) *Raf*	Christmas Vine	Italy	2	Isor, Kaem, Kaem-8-ME, Kaem-7-O-rham, Quer	CA, FA, p-CouA	VA	N.I	N.I	9	[30,79]
39.	Cucurbitaceae	*Cucumis melo*	Honeydew	China	1	Chr, Kaem, Querc, Hest,	CA	p-HBA, ProA	Prod	N.I.	8	[43]
40.	Cucurbitaceae	*Cucurbita* sp.	Squash Blossoms	Turkey	1	Api, Chr, Lut, Kaem, Rut, Hest, Nar, CG	CA, FA, o-CouA,	ElA, GA, GenA, ProA, p-HBA, SyrA, VA	HGA, Prod, Van	N.I.	21	[53]
41.	Cunoniaceae	*Eucryphia cordifolia Cav.*	Ulmo	Chile	1	Api, Chr, Quer, Pinoc	CA, ChloA, p-CouA	GA, m-HBA,	N.I.	AbsA	10	[80]
42.	Cunoniaceae	*Weinmannia racemosa*	Kamahi	China, New Zealand	2	Api, Lut, Gal, Kaem, Quer, Querc, Hest, Nar	CA, ChloA	2,3,4-THBA, GA, GenA, SyrA	Prod, Van	Leptd	17	[43,81]
43.	Ericaceae	*Arbutus unedo* L., *Arbutus unedo*	Strawberry Tree, Arbousie, Arbutus	Italy, Morocco	4	Api, Lut, Gal, Isor, Kaem, Myr, Quer, Rut, Pinoc, Pinob, EC	CA, FA, p-CouA, RosA, t-CA,	GA, p-HBA, SyrA	HGA	AbsAa	21	[22,34,40,42]
44.	Ericaceae		Calluna, Heather, Erica, Bell Heather, Ling Heather	Algeria, Bulgaria, Finland, France, Germany, Italy, New Zealand, Poland, Portugal, Spain, Turkey	24	Api, Chr, Lut, Gal, Isor, Kaem, Kaem-8-ME, Kaemf, Myr, Myr-3-ME, Quer, Quer-rham, Querc, Rham, Rut, Hest, Isosak, Nar, Pinoc, Pinob, C, CG, Dai, Gene	CA, CADAE, CAPE, ChloA, FA, m-CouA, o-CouA, p-CouA, RosA, t-CA	BenA, ElA, GA, GenA, MS, m-HBA, ProA, p-HBA, ResA, SalA, SyrA, VA	3,4-DHPAA, HGA, HVA, PAA, p-HPAA, DL-p-HPLA, DL-β-PLA, 3-PPA, Prod, Van	AbsA, Lum	58	[33,36,46,47,49,53,56,59,61,70,82,83,84,85,86,87,88,89,90]
45.	Ericaceae	*Oxydendrum arboretum*	Sourwood	Malaysia	1	Hest	CA	N.I.	N.I.	N.I.	2	[77]
46.	Ericaceae	*Rhododendron ponticum*	Rhododendron	France, Italy, Portugal, Spain, Turkey	9	Aca, Api, Chr, Lut, Isor, Kaem, Kaem-8-ME, Quer, Rut, Hest, Nar, Pinoc, Pinob, C, EC, Gene	CA, CADAE, CAPE, ChloA, FA, m-CouA, o-CouA, p-CouA, t-CA	BenA, GA, GenA, ProA, p-HBA, ResA, SyrA, VA	HGA, Prod	N.I.	35	[36,46,47,51,53,61,87,88,91]
47.	Ericaceae	*Vaccinium* sp. *V. vitis-idaea*	Mire Lingonberry	Finland	3	Aca, Kaem-7-O-rham, Rham, Nar-ME, GC	ChloA, FA, p-CouA, t-CA	BenA, ProA, p-HBA, VA	3-PPA	N.I	14	[89,92]
48.	Euphorbiaceae	*Croton* sp.	Morrão de Candeia	Brazil	1	Gal, Nar	ChloA, m-CouA	BenA, GA, p-HBA, SyrA	N.I.	AbsA	9	[45]
49.	Euphorbiaceae	*Euphorbia* sp.	Euphorbia, Spurge, Daghmos	Morocco, Turkey	2	Api, Chr, Lut, Kaem, Rut, Hest, Nar, CG, EC	CA, FA, o-CouA, p-CouA	ElA, GA, GenA, ProA, p-HBA, SyrA, VA	HGA, Prod	N.I.	22	[34,53]
50.	Euphorbiaceae	*Hevea brasiliensis*	Rubber Tree	Malaysia	1	Myr, C	CA	BenA, GA	N.I.	N.I.	5	[77]
51.	Fabaceae	*Acacia catechu*	Acacia	Lithuania	1	N.I.	CA, ChloA, FA, RosA, t-CA,	N.I.	N.I.	N.I.	5	[93]
52.	Fabaceae	*Acacia ehrenbergiana*	Acacia	Saudi Arabia	1	Api, Chr, Lut, Gal, Quer	CA, ChloA, p-CouA	GA, p-HBA, SyrA	p-HPAA		12	[41]
53.	Fabaceae	*Acacia mangium*	Acacia	Malaysia	5	Kaem, Quer, Rut, Hest, Nar, C	CA, ChloA, FA, p-CouA, t-CA	BenA, ElA, PGG, SyrA	N.I.	N.I.	15	[76,77,94]
54.	Fabaceae	*Acacia* sp.	Acacia, Acacia Flower	China	3	Lut, Quer Querc, Hest, Narg	N.I.	2,3,4-THBA, GA, GenA, p-HBA	Prod	N.I.	10	[43]
55.	Fabaceae	*Acacia tortilis*	Acacia, Wild Mountain, Oman Same, Rasul Khaima Samar, Doany Samer, Marya Herba, Ashab Marya Samer	UAE, Saudi Arabia	5	Api, Chr, Gal, Kaem, Myr, Quer, Rut, Nar, Narg, C, EC	CA, ChloA, FA, p-CouA, t-CA	GA, p-HBA, SyrA, VA	p-HPAA	N.I.	21	[41,95]
56.	Fabaceae	*Astragalus membranaceus (Fisch.) Bunge,* *A.microcephalus Willd.*, *A. sinicus*	Astragalus	China, Turkey	3	Api, Bai, Chr, Lut, Vit, Kaem, Myr, Quer, Querc, Rut, Hest, Hesd, Nar, Pinoc, Cal, Form, Gene, Ono, Cal-7-O-β-D-gluc	CA, ChloA, FA, IfA, p-CouA, SinA	BenA, SalA, m-HBA, p-HBA, ProA, VA, GA, SyrA	N.I.	AbsA	34	[36,62,96]
57.	Fabaceae	*Ceratonia siliqua*	Carob	Turkey	1	Api, Chr, Lut, Kaem Rut, Hest, Nar, Gene	CA, FA, o-CouA, p-CouA	GA, GenA, ProA, p-HBA, SyrA, VA	HGA, Van, Prod	N.I.	21	[53]
58.	Fabaceae	*Glycine max*	Soybean	USA	1	N.I.	p-CouA, t-CA,	p-HBA	N.I.	N.I.	3	[32]
59.	Fabaceae	*Hedysarum* sp., *H. coronarium*, *H. coronarium,* L.	Hedysarum Sulla, Fior Di Sulla	Algeria, Bulgaria Italy	10	Api, Chr, Lut, Gal, Isor, Kaem, Myr, Quer, Rut, Hest, Hesd, Isosak, Nar, Pinoc, Pinob, C, EC	CA, ChloA, FA, o-CouA, p-CouA, t-CA,	BenA, GA, GenA, ProA, p-HBA, SalA, SyrA, VA	PAA, p-HPAA, DL-p-HPLA, 5,7-DMCoum	AbsA, PhAn, Tyr	38	[33,42,44,56,59,61,86,97,98,99]
60.	Fabaceae	*Lotus* sp.	Lotus	Argentina, Algeria	2	Api, Chr, Lut, Gal Isor, Kaem, Myr, Quer, Pinob	CA, FA, p-CouA	BenA, p-HBA, SyrA, VA	p-HPAA	N.I.	17	[33,100]
61.	Fabaceae	*Lysiloma latisiquum* (L.) *Benth*	Singing Bean	Italy	1	Isor, Kaem, Kaem-7-O-rham, Kaem 8-ME, Myr, Quer, Quer-diGlc, Quer-rham	CA, p-CouA	VA	N.I.	N.I.	11	[30]
62.	Fabaceae	*Medicago sativa*	Alfalfa, Lucerne	Argentina, Spain	2	Aca, Api, Chr, Gal, Isor, Kaem, Quer, Quer-3-ME, Isosak, Pinoc, Pinob	CA, CAPE, FA, o-CouA,	ElA, SyrA	N.I.	N.I.	17	[51,101]
63.	Fabaceae	*Melilotus officinalis* L., *Melilotus* sp.	Melilotus, Yellow Sweet Clover, Clover	Algeria, Poland, USA	3	Api, Chr, Lut, Gal, Isor Kaem, Mor, Myr, Quer, Pinoc, Pinob, Diadzein, Gene, C	CA, FA, p-CouA, RosA, t-CA	BenA, ElA, GA, m-HBA, p-HBA, SyrA, VA	p-HPAA	N.I.	27	[32,33,60]
64.	Fabaceae	*Prosopis nigra*, *P. juliflora*	Algarrobo, Ghaf	Argentina, UAE	2	Chr, Hest, Pinoc	FA, p-CouA, t-CA	SyrA	N.I.	N.I.	7	[95,102]
65.	Fabaceae	*Robinia pseudoacacia* L.	Acacia, Black Locust, Acacia grove, Robinia	Austria, Belgium, Bulgaria, China, Croatia, Czech Republic, Italy, Poland, Portugal, Romania, Serbia, Slovakia, Slovenia, Spain, Switzerland, Turkey, USA	36	Aca, Api, Bai, Chr, Chr-2′-ME, Genk, Lut, Tec, Vit, Fis, Gal, Isor, Kaem, Kaem-8-ME, Kaem-7-O-rham, Kaem-3-O-(6”-acetyl)-β-Gluc, kaem-3-O-(hexoxyl) rob-7-O-rham, kaem-3-O-(hexoxyl) robi, kaem-3-O-hex-7-O-rham, kaem-3-O-rob-7-O-rham, kaem-3-O-rob, Mor, Myr, Quer, Quer-3,3-DME, Quer-3,7-DME, Quer-3-ME, Querc, Rham, Rut, Alp, Erio, Hest, Hesd, Isosak, Nar, Pinoc, Pinos, Sak, Pinob, Pinob-3-O-ace, Pinob-5-ME, Tax, CG, EGC, GC, Cal, Form, Gene, Geni, Ono, Cal-7-O-β-D-gluc, Pinob Chal	3,4-DMCA, CA, CABE, CADAE, CAPE, ChloA, CChloA, FA, IfA, m-CouA, o-CouA, p-CouA, RosA, SinA, t-CA	BenA, CuA, ElA, GA, GenA, MS, m-HBA, ProA, p-HBA, SalA, SyrA, VA	HGA, PAA, DL-β-PLA, 3-PPA, 5-Phenylpent-4-enoic acid, 2-M-4-VP, 2,3,5-TMP, 2-MBd, Prod, Van, 5,7-DMCoum, DBZO	AbsA	93	[32,35,36,37,44,46,49,50,51,53,54,56,57,59,61,62,63,64,66,67,70,71,72,86,96,97,103,104,105,106,107,108,109,110]
66.	Fabaceae	*Trifolium repems*, *Trifolium* sp., *Trifolium pratense*	Clover, Trifolium, Trefoils, 45° South clover	Algeria, Argentina, Austria, China, Germany, Italy, New Zealand, Turkey	12	Api, Chr, Lut, Gal, Isor, Kaem, Quer, Rut, Hest, Isosak, Nar, Pinoc, Pinob, Gene	CA, ChloA, FA, o-CouA, p-CouA, SinA, t-CA	3,4,5-TMBA, BenA, ElA, GA, GenA, M-4-HBz, MS, OAA, PAA, ProA, p-HBA, SalA, SyrA, VA, VAME	3,4-DHPAA, HGA, HVA, PAA, p-HPAA, 4-mPLA, DL-β-PLA, 3-PPA, PhlA, 4-MPC, Prod, Van	AbsA, Leptd	50	[33,36,43,48,53,56,57,81,82,101,111,112]
67.	Fabaceae	*Vicia dichroantha*, *V. villosa Roth*	Vicia	China	2	Gal, Kaem, Quer, Rut, GC	CA, ChloA, FA, p-CouA, RosA	BenA, p-HBA, ProA, GA, SyrA, ElA	N.I	N.I	16	[68,96]
68.	Fagaceae	*Castanea sativa Mill.*, *C. sativa Miller*	Chestnut	Austria, Belgium, Bulgaria, France, Germany, Italy, Portugal, Slovenia, Spain, Switzerland, Turkey	28	Aca, Api, Chr, Chr-2′-ME, Chr-6-ME, Genk, Lut, Tec, Gal, Gal-5-ME, Isor, Kaem, Kaem-ME, Kaem-8-ME, Myr, Quer, Quer-3,3-DME, Quer-3,7-DME, Quer-3-ME, Querc, Rham, Rut, Hest, Nar, Pinoc, Pinob, pinob-3-O-pent, Pinob-5-ME, C, CG, EC, Gene, Leptosin, Pinob Chal	3,4-DMCA, CA, CAIPE, CAPE, ChloA, FA, m-CouA, o-CouA, p-CouA, SinA, t-CA	BenA, CuA, ElA, GA, GenA, M-4-HBz, MS, m-HBA, OAA, ProA, p-HBA, ResA, SalA, SyrA, VA, VAME	HGA, PAA, DL-p-HPLA, DL-β-PLA, 3-PPA, 5-Phenylpent-4-enoic acid, 2-M-4-VP, 2-MBd, Prod, Van, 1-(2-Aminophenyl)butan-1-one, 5,7-DMCoum, DBZO	KyA, Lum	76	[35,36,40,46,47,49,51,53,56,57,59,61,86,87,88,90,97,98,104,109,113,114,115,116,117,118,119]
69.	Fagaceae	*Fagus* sp.	Beech Forest	China	1	Api, Lut, Quer, Querc	N.I.	2,3,4-THBA, GA, GenA, ProA, p-HBA	Van, Prod	N.I.	11	[43]
70.	Fagaceae	*Quercus* sp., *Q. robur* L.	Oak	Switzerland, Turkey	4	Aca, Chr, Chr-2′-ME, Genk, Tec, Gal, Quer, Rut, Pinoc, Pinob-5-ME, EC, Pinob Chal	3,4-DMCA, CA, FA, p-CouA, t-CA,	BenA, CuA, GA, MS, ProA, p-HBA, SalA, SyrA	3-PPA, 5-Phenylpent-4-enoic acid, 2-M-4-VP, 2-MBd, DBZO,	N.I.	30	[36,88,104,120]
71.	Hydrophyllaceae	*Phacelia tanacetifolia*	Phacelia	Poland	1	Api, Chr, Gal, Kaem, Myr, Quer Nar, Pinoc, EC	CA, ChloA, FA, p-CouA, t-CA,	GA, p-HBA	N.I.	AbsA	17	[121]
72.	Hypericaceae	*Hypericum* sp.	Hypericum	China	1	Lut, Kaem, Quer, Narg	CA, ChloA,	GA, p-HBA	Prod	N.I.	9	[43]
73.	Iridaceae	*Crocus sativus*	Saffron	India	1	Api, Kaem, Myr, Quer, Nar, Pinob, C	CA, ChloA, FA, p-CouA	GA, ElA	N.I.	N.I.	13	[122]
74.	Lamiaceae	*Agastache* sp.	Agastache	Australia	1	Kaem, Quer, Rut, Hest	CA, ChloA, FA, p-CouA, SinA, t-CA,	MS, ProA, p-HBA, ResA, SyrA, VA,	PAA, DL-β-PLA	N.I.	18	[123]
75.	Lamiaceae	*Lavandula* sp. *L. stoeclias*	Lavender	Bulgaria, France, Hungary, Italy, Portugal, Spain, Switzerland, Turkey	10	Aca, Api, Chr, Chr-2′-ME, Genk, Lut, Tec, Gal, Isor, Kaem, Kaem-8-ME, Myr, Quer, Quer-3-ME, Rut, Erio, Hest, Nar, Pinoc, Pinob, Pinob-5-ME, C, CG, EC, Pinob Chal	3,4-DMCA, CA, CADAE, CAPE, ChloA, FA, m-CouA, o-CouA, p-CouA, RosA, t-CA	BenA, CuA, ElA, GA, GenA, MS, ProA, p-HBA, SalA, SyrA, VA	HGA, PAA, DL-p-HPLA, DL-β-PLA, 3-PPA, 5-Phenylpent-4-enoic acid,2-M-4-VP, 2-MBd, Prod, Emo, n-β-L, DBZO	AbsA	60	[36,40,46,47,49,51,53,59,104,124]
76.	Lamiaceae	*Leonurus cardiaca*	Motherwort	China	1	N.I.	GA, p-HBA	N.I.	Prod, Van	N.I.	4	[43]
77.	Lamiaceae	*Ocimum basilicum*	Basil	Serbia	1	Api, Chr, Lut, Gal, Kaem, Quer, Rut, Pinoc	CA, ChloA	GA, ProA	N.I.	N.I.	12	[54]
78.	Lamiaceae	*Phlomis armeniaca Willd.*	Jerusalem Tea	Turkey	1	Api, Quer	ChloA, FA, p-CouA	p-HBA, VA	N.I.	N.I.	7	[36]
79.	Lamiaceae	*Plectranthus rugosus*	Wild Bush	India	1	Api, Kaem, Myr, Quer, Nar, Pinob, C	CA, ChloA, FA, p-CouA	ElA, GA	N.I.	N.I.	13	[122]
80.	Lamiaceae	*Prunella vulgaris*	Prunella	China	1	Api, Chr, Gal, Quer, Nar, Pinob	CA, ChloA, FA, p-CouA, RosA, t-CA,	ElA, ProA, SyrA, VA,	N.I.	N.I.	16	[125]
81.	Lamiaceae	*Rosmarinus officinalis* L.	Rosemary	Bulgaria, Czech Republic, France, Italy, Portugal, Spain, Tunisia	13	Aca, Api, Chr, Chr-6-ME, Lut, Tec, Gal, Gal-5-ME, Isor, Kaem, Kaem-ME, Kaem-8-ME, Kaemf, Myr Myr-3,7,4′5′-TeME, Quer Quer-3,3-DME, Quer-3,7-DME, Quer-7,3′-DME, Rham, Rut, Erio, Nar, Pinoc, Pinos, Sak, Pinob, Pinob-3-O-butyr, Tax, C, Gene	CA, CAIPE, CAPE, ChloA, FA, IfA, p-CouA, RosA, SinA, t-CA	GA, GenA, m-HBA, ProA, p-HBA, SalA, SyrA, VA	PAA, DL-β-PLA, 5,7-DMCoum	N.I.	52	[10,40,46,47,49,51,52,59,86,107,116,126,127]
82.	Lamiaceae	*Salvia officinalis* L.	Sage	Croatia	2	Api, Chr, Lut, Gal, Isor, Kaem, Quer, Hest, Nar, Pinoc, Pinos, Pinob, EC, EGC, EGCG, GC, GCG	CA, ChloA, FA, p-CouA, RosA	GA, GenA, ProA, p-HBA	Resv	N.I.	27	[128,129]
83.	Lamiaceae	*Satureja hortensis*, *Satureja subspicata Vis.*	Savory, Satureja	Italy, Croatia	2	Api, Chr, Gal, Kaem, Quer, Gene	ChloA, p-CouA	BenA, GA, MS	DL-β-PLA	PhAn, Tyr	14	[97,130]
84.	Lamiaceae	*Sideritis* sp.	Sideritis	Turkey	1	Api, Chr, Lut, Kaem, Rut, Hest, Nar, CG	CA, FA, o-CouA	ElA, GA, GenA, ProA, p-HBA, SyrA, VA	HGA, Prod, Van	N.I.	21	[53]
85.	Lamiaceae	*Thymus* sp., *T. algeriensis*, *T. capitatus* (L.), *T. capitatus and T. herba-borona,* *T. capitatus Hoffgg.e.LK.,* *T. vulgare,* *T. vulgaris* L., *T. capitatus*	Thyme /Zaˆatar /Zohif	Belgium, China, Greece, Hungary, Italy, Morocco, Portugal, Tunisia, Turkey	16	Api, Chr, Chr-6-ME, Lut, Gal, Gal-5-ME, Isor Kaem, Kaem-ME, Kaem-8-ME, kaem-3-O-neoh, Myr, Myr 3,7,4′5′-TeME, Quer, Quer-3,3-DME, Quer-3,7-DME, Quer-3-ME, Querc, Rham, Rut, Erio, Hest, Nar, Pinoc, Pinos, Pinob, CG, EC, Gene	CA, CADAE, CAPE, ChloA, FA, Gene, o-CouA, p-CouA, RosA, t-CA	ElA, GA, GenA, ProA, p-HBA, ResA, SyrA, VA	HGA, Prod, Van	KyA	51	[34,35,40,43,46,52,53,61,97,116,124,131,132,133,134]
86.	Lamiaceae	*Vitex agnus-castus* L.	Chaste	China, Turkey	3	Api, Chr, Lut, Gal, Kaem, Myr, Quer, Rut, Pinoc, C	3,4-DMCA, CA, ChloA, FA, p-CouA, RosA, SinA, t-CA	ElA, GA, ProA, p-HBA, ResA, VA	N.I.	N.I.	24	[36,64,135]
87.	Lamiaceae	*Vitex negundo var. heterophylla Rehd.*	Vitex	China, Turkey	3	Api, Bai, Chr, Lut, Vit, Fis, Gal, Isor, Kaem, Mor, Quer, Querc, Rut, Hest, Hesd, Isosak, Nar, Pinoc, Sak, Pinob, Tax, EGC Form, Gene, Geni, Ono,	CA, ChloA, CChloA, FA, IfA, o-CouA, p-CouA, SinA	GA, GenA, m-HBA, ProA, p-HBA, SalA, SyrA, VA	HGA, Prod, Van	AbsA	46	[37,53,62]
88.	Lauraceae	*Persea americana*	Avocado	Spain	1	Chr, Gal, Gal-5-ME, Isor, Kaem, Kaem-3-O-neoh, Quer, Quer-3,7-DME, Rut, Pinoc, Pinob, Pinob-5-ME	N.I.	ElA	N.I.	N.I.	13	[116]
89.	Malvaceae	*Gaya macrantha*	Field Flower	Brazil	1	N.I.	N.I.	GA	N.I.	N.I.	1	[31]
90.	Malvaceae	*Gossypium hirsutum* L.	Cotton	Turkey	1	Api, Chr, Lut, Kaem, Rut, Hest, Nar, CG	CA, FA, o-CouA	ElA, GA, GenA, ProA, p-HBA, SyrA, VA	HGA, Prod,	N.I.	20	[53]
91.	Malvaceae	*Tilia* sp., *T. amurensis Rupr.*, *T. argentea*, *T. cordata*, *T. cordata* L., *T. europa*, *T. europaea*, *T. scop*, *T. platyphyllos*	Linden, Tilia, Lime Tree, Lime, Lime-blossom, Linden tree, Linden blossom	Austria, Bulgaria, China, Czech Republic, France, Germany, Italy, Poland, Romania, Serbia, Slovenia, Spain, Turkey	25	Aca, Api, Bai, Chr, Lut, Tec, Vit, Fis, Gal, Isor, Kaem, Kaem-8-ME, Kaemf, Mor, Myr, Quer, Quer-3-ME, Querc, Rham, Rut, Hest, Hesd, Isosak, Nar, Narg, Pinoc, Sak, Pinob, Pinob-3-O-ace, Tax, C, EGC, Form, Gene, Ono	CA, CAPE, ChloA, CChloA, FA, IfA, p-CouA, p-MCA, RosA, SinA, t-CA	BenA, ElA, GA, GenA, M-4-HBz, MS, m-HBA, ProA, p-HBA, SalA, SyrA, VA, VAME	3,4-DHPAA, HGA, MandA, PAA, p-HPAA, DL-β-PLA, 3-PPA, 4-MPC, Prod	AbsA	69	[36,37,43,47,48,49,50,51,54,57,59,61,62,65,66,68,70,72,107,109,110,114,136]
92.	Meliaceae	*Azadiractha indica*	Neem	India	1	Lut, Isor Myr Quer Rut, C, EC	p-CouA, CA, FA, ChloA	ProA, GA, SyrA	N.I.	N.I.	14	[137]
93.	Myrtaceae	*Eucalyptus camaldulensis*	River Red Gum Eucalyptus	Australia, Italy, Spain, Switzerland	4	Aca, Api, Chr, Chr-2′-ME, Genk, Lut, Tec, Tri, Gal, Kaem, Myr, Quer, Pinoc, Pinob, Pinob-5-ME, GC	3,4-DMCA, CA, ChloA, FA, p-CouA, t-CA	BenA, GA, p-HBA, SalA, SyrA	PAA, 2-M-4-VP, 2,3,5-TMP, 2-MBd, 1-(3-methoxy-phenyl) ethanone, DBZO	AbsA	35	[40,104,138,139]
94.	Myrtaceae	*Eucalyptus crebra*	Narrow-leaved Ironbark	Australia	1	N.I.	CA, ChloA, FA, p-CouA,	ElA, GA	N.I.	AbsA	7	[139]
95.	Myrtaceae	*Eucalyptus globoidea*	Stringybark	Australia	1	N.I.	CA, ChloA, p-CouA	ElA, GA	N.I.	AbsA	6	[139]
96.	Myrtaceae	*Eucalyptus globulus*	Eucalyptus	Lithuania	1	N.I.	CA, ChloA, FA, RosA, t-CA	GA, VA	N.I.	N.I.	7	[93]
97.	Myrtaceae	*Eucalyptus intermedia*	Bloodwood	Australia	1	N.I.	CA, ChloA, FA, p-CouA	ElA, GA	N.I.	AbsA	7	[139]
98.	Myrtaceae	*Eucalyptus largiflorens*	Blackbox	Australia	1	N.I.	CA, ChloA, FA, p-CouA	ElA, GA	N.I.	AbsA	7	[139]
99.	Myrtaceae	*Eucalyptus marginata*	Jarrah	Australia	1	Quer, Rut, Hest	CA, ChloA, FA, p-CouA, SinA, t-CA,	GA, MS, ProA, p-HBA, SyrA, VA	DL-β-PLA	N.I.	16	[123]
100.	Myrtaceae	*Eucalyptus melliodora*	Yellow Box	Australia, Spain	2	Lut, Tri, Kaem, Myr, Quer, Quer-3-ME	CA, ChloA, FA, p-CouA	ElA, GA	N.I.	AbsA	13	[138,139]
101.	Myrtaceae	*Eucalyptus moluccana*	Gum Top	Australia	1	N.I.	CA, ChloA, FA, p-CouA	ElA, GA	N.I.	AbsA	7	[139]
102.	Myrtaceae	*Eucalyptus nubila*	Blue Top Ironbark	Australia	1	N.I.	CA, ChloA, FA, p-CouA	ElA, GA	N.I.	AbsA	7	[139]
103.	Myrtaceae	*Eucalyptus ochrophloia*	Yapunyah	Australia	1	N.I.	CA, ChloA, FA, p-CouA	GA, ElA	N.I.	AbsA	7	[139]
104.	Myrtaceae	*Eucalyptus pilligaensus*	Mallee	Spain	1	Lut, Tri, Myr, Quer, Pinob, Pinoc, GC	N.I.	N.I.	N.I.	N.I.	7	[138]
105.	Myrtaceae	*Eucalyptus* sp.	Eucalyptus	Algeria, Argentina, Belgium, Brazil, Bulgaria, China, Germany, India, Italy, Spain, Tunisia, Turkey	21	Api, Chr, Chr-6-ME, Lut, Tec, Tri, Gal, Gal-5-ME, Isor, Kaem, Kaem-8-ME, kaem-3-O-neoh, Kaemf, Myr, Myr-3,7,4′5′-TeME, Quer, Quer-3,3-DME, Quer-3,7-DME, Quer-3-ME, Quer-3-O-hex (1→2) hex, Quer-rham, Rham, Rut, Hest, Isosak, Nar, Narg, Pinoc, Pinos, Pinob, C, EC, Dai, Gene, Leptosin	3,4-DMCA, CA, ChloA, FA, m-CouA, o-CouA, p-CouA, SinA, t-CA	2,3,4-THBA, BenA, ElA, GA, GenA, MS, m-HBA, ProA, p-HBA, SalA, SyrA, VA	3,4-DHPAA, HGA, HVA, PAA, p-HPAA, DL-p-HPLA, DL-β-PLA, Prod, Van, 5,7-DMCoum	KyA, Lum, PhAn, Tyr	70	[31,33,35,42,43,49,51,52,53,59,61,64,86,90,98,100,101,114,116,137,138]
106.	Myrtaceae	*Kunzea ericoides*	Kanuka	Germany, New Zealand	5	Leptosin	N.I.	3,4,5-TMBA, GA, Lepp, MS, OAA, PAA, SyrA	4-mPLA, DL-p-HPLA, DL-β-PLA, 3,4,5-TMP, p-And, 2′-MAPo	5-MF-3-CA, AbsA, KojA, Leptd, Lum	19	[81,111,112,140,141]
107.	Myrtaceae	*Miellerie, Leptospermum lanigerum, and Leptospermum scoparium*	Tea tree	Australia	1	Kaem, Quer, Rut, Hest	CA, ChloA, FA, p-CouA, SinA, t-CA	p-HBA, ProA, VA, GA, SyrA, ResA, MS	DL-β-PLA	N.I.	18	[123]
108.	Myrtaceae	*Leptospermum polygalifolium*	Jelly Bush	Australia, Germany, New Zealand	4	Chr, Lut, Tec, Tri, Isor, Kaem, Kaem-8-ME, Quer, Quer-3,3-DME, Quer-3-ME, Rut, Hest, Pinoc, Pinob, Leptosin	CA, ChloA, FA, p-CouA, SinA, t-CA	GA, MS, OAA, ProA, p-HBA, ResA, SyrA, VA	DL-β-PLA, 3,4,5-TMP, 2’-MAPo	5-MF-3-CA, KojA, Leptd	35	[112,123,141,142]
109.	Myrtaceae	*Leptospermum* *scoparium*	Manuka	Australia, China, Germany, Italy, Malaysia, New Zealand, Poland, Spain, Thailand, UAE, USA	29	Api, Chr, Chr-6-ME, Lut, Vit, Fis, Gal, Isor, Kaem, Kaem-8-ME, Myr, Quer, Quer-3,3-DME, Quer-3,7-DME, Quer-3-ME, Quer-3-O-hex (1→2) hex, Querc, Rut, Hest, Hesd, Isosak, Nar, Narg, Pinoc, Sak, Pinob, Tax, C, EC, GC, Form, Leptosin	CA, CAPE, ChloA, CChloA, FA, IfA, p-CouA, RosA, SinA, t-CA	2,3,4-THBA, 3,4,5-TMBA, BenA, ElA, GA, GenA, Lepp, MS, OAA, PAA, ProA, p-HBA, ResA, SalA, SyrA, VA	PAA, p-HPAA, 4-mPLA, DL-p-HPLA, DL-β-PLA, 3,4,5-TMP, p-And, Prod, 2’-HAPo, 2′-MAPo, 3-hydroxy-1-(2-methoxyphenyl)penta-1,4-dione	2-MBF, 5-MF-3-CA, AbsA, KojA, Leptd, Lum,	75	[37,38,43,81,82,84,90,95,111,112,116,123,140,141,142,143,144,145,146,147,148]
110.	Myrtaceae	*Lophostemon conferta*	Brush Box	Australia	1	N.I.	CA, ChloA, FA, p-CouA	ElA, GA, SyrA,	N.I.	AbsA	8	[58]
111.	Myrtaceae	*Melaleuca cajuputi*	Gelam	Malaysia	7	Api, Chr, Lut, Kaem, Myr, Quer, Rut, Hest, Nar, Pinob-3-O-prop	CA, ChloA, FA, p-CouA, t-CA	BenA, GA, SyrA, ElA	N.I.	N.I.	19	[39,75,76,77,94,149]
112.	Myrtaceae	*Melaleuca quinquenervia*	Tea Tree	Australia	1	N.I.	CA, ChloA, FA, p-CouA	ElA, GA, SyrA	N.I.	AbsA	8	[58]
113.	Myrtaceae	*Metrosideros robusta*	Rata	China	1	Api, Chr, Lut, Quer, Querc, Hest	CA, ChloA	2,3,4-THBA, GA, SyrA	Prod	N.I.	12	[43]
114.	Myrtaceae	*Myrtaceae* sp.	Myrtaceae	Algeria	1	Api, Chr, Lut, Gal, Isor, Kaem, Kaemf, Myr, Quer, Isosak, Pinoc, Pinob	t-CA, p-CouA, CA, FA	BenA, GenA, ProA, p-HBA, SyrA, VA	3,4-DHPAA, p-HPAA	N.I.	24	[33]
115.	Myrtaceae	*Myrtus communis* L.	Myrtus	Italy	1	Api, Lut, Gal, Quer, Pinoc, Pinob, C	t-CA	N.I.	N.I.	N.I.	6	[40]
116.	Nelumbonaceae	*Nelumbo nucifera*	Padma Flower	Bangladesh	1	Kaem, C	CA, FA, t-CA	N.I.	N.I.	N.I.	5	[69]
117.	Nothofagaceae	*Nothofagus* sp.	Beech	New Zealand	1	Chr, Gal, Pinoc, Pinob	p-CouA, t-CA	BenA, MS, p-HBA, SyrA, VA	N.I.	N.I.	11	[82]
118.	Nyssaceae	*Nyssa aquatica*	Tupelo	USA	2	Chr, Gal, Kaem, Quer, Hest, Pinoc	p-CouA, t-CA	VA	N.I.	N.I.	9	[32,38]
119.	Oleaceae	*Osmanthus fragrans*	Wild Osmanthus	China	1	N.I.	N.I.	2,3,4-THBA, GenA, SyrA, p-HBA	HGA, Prod	N.I.	6	[43]
120.	Onagraceae	*Epilobium angustifolium*	fireweed willow herb	Finland, USA	2	N.I.	p-CouA, t-CA	BenA, p-HBA	3-PPA	N.I.	5	[32,89]
121.	Pedaliaceae	*Sesamum indicum*	Teel/Sesame	Bangladesh	1	Gal, Nar, C	CA, ChloA, FA	GA	N.I.	N.I.	7	[69]
122.	Pinaceae	*Abies* sp., *A. alba* Mill., *A. cephallonica*, *A. cephalonica* Loudon	Fir	France, Greece, Slovenia	5	Api, Chr, Lut, Gal, Isor, Kaem, Kaem-8-ME, Myr, Quer, Nar, Pinoc, Pinob	CA, FA, p-CouA	ProA, p-HBA, SyrA, VA	N.I.	N.I.	19	[47,109,132,133,134]
123.	Pinaceae	*Cedrus libani var. stenocoma*	Cedar	Turkey	1	Api, Chr, Lut, Kaem, Rut, Hest, Nar, Gene	CA, FA, o-CouA, p-CouA, t-CA	GA, GenA, ProA, p-HBA, SyrA, VA	HGA, Van, Prod	N.I.	22	[53]
124.	Pinaceae	*Picea abies* (L) *Karst*	Spruce	Slovenia	1	Api, Chr, Lut, Gal, Kaem, Myr, Quer, Nar, Pinoc, Pinob	N.I.	N.I.	N.I.	N.I.	10	[109]
125.	Pinaceae	*Pinus* sp., *Pinus brutia* L.	Forest Fino Pine	Greece, Turkey	6	Api, Chr, Lut, Kaem, Myr, Quer, Rut, Hest, Nar, Pinob, C, CG, Gene	o-CouA, p-CouA, CA, FA	p-HBA, ProA, GA, GenA, SyrA, VA	HGA, Prod, Van	N.I.	26	[36,53,132,133,134]
126.	Polygonaceae	*Fagopyrum esculentum*	Buckwheat	China, Finland, Italy, Lithuania, Poland, Serbia, Turkey, USA	16	Api, Chr, Lut, Vit, Fis, Gal, Isor, Kaem, Mor, Myr, Quer, Rut, Hest, Hesd, Isosak, Nar, Pinoc, Sak, Pinob, Tax, Form, GC	CA, CAPE, ChloA, CChloA, FA, IfA, RosA, SinA, p-CouA, t-CA	BenA, ElA, GA, m-HBA, ProA, p-HBA, SalA, SyrA, VA,	HGA, 3-PPA	AbsA	44	[32,37,54,55,66,70,73,83,84,88,89,93,145,150,151,152]
127.	Proteaceae	*Banksia ericifolia*	Heath	Australia	1	N.I.	CA, ChloA, FA, p-CouA	ElA, GA	N.I.	AbsA	7	[58]
128.	Proteaceae	*Knightia excelsa*	Rewarewa	New Zealand	2	N.I.	N.I.	3,4,5-TMBA, GA, MS, OAA, PAA, SyrA	4-mPLA, DL-β-PLA	AbsA, Leptd	10	[81,111]
129.	Ranunculaceae	*Coptis* sp.	Mountain Coptis	China	1	Lut, Gal, Kaem, Quer	ChloA	p-HBA, GA, SyrA	Prod	N.I.	9	[43]
130.	Ranunculaceae	*Nigella sativa*	Kalijira	Bangladesh	1	Nar, C	CA, FA, ChloA	N.I.	N.I.	N.I.	5	[69]
131.	Rhamnaceae	*Frangula* sp.	Alder	France	1	Kaem-8-ME	N.I.	N.I.	N.I.	N.I.	1	[47]
132.	Rhamnaceae	*Gouania polygama (Jack)Urb*	Linen vine	Italy	1	Isor, Kaem, Kaem-8-ME, Kaem-7-O-rham, Quer Quer-rham	CA, FA, p-CouA	SyrA, VA	N.I.	N.I.	11	[30]
133.	Rhamnaceae	*Hovenia dulcis*	Japanese grape	Brazil	1	Quer	p-CouA	GA	N.I.	N.I.	3	[31]
134.	Rhamnaceae	*Paliurus spina-christi*	Marruca	Italy	1	Quer, Hesd, Nar	CA, FA, p-CouA	p-HBA, VA	N.I.	N.I.	8	[56]
135.	Rhamnaceae	*Ziziphus jujuba*, *Ziziphus jujube Mill*	Jujube/Zaohua	China	4	Api, Chr, Lut, Vit, Gal, Isor, Kaem, Myr, Quer, Hest, Isosak, Nar, Pinoc, Sak, Pinob, Tax, GC	CA, ChloA, CChloA, FA, IfA, p-CouA, RosA, SinA, t-CA	BenA, ElA, GA, GenA, ProA, p-HBA, ResA, SalA, SyrA, VA	Prod	N.I.	37	[37,43,96,135]
136.	Rhamnaceae	*Ziziphus spina-csisti*	Wild jujube	Morocco, Saudi Arabia UAE	7	Api, Chr, Gal, Kaem, Myr, Quer, Rut, Nar, Narg, C, EC	CA, ChloA, FA, p-CouA, RosA, t-CA	GA, p-HBA, SyrA, VA	p-HPAA	N.I.	22	[34,41,95]
137.	Rosaceae	*Crataegus* sp.	Wild hawthorn	China	1	N.I.	N.I.	GA, GenA, ProA, p-HBA	Prod	N.I.	5	[43]
138.	Rosaceae	*Eriobotrya japonica*	Loquat	China	1	N.I.	N.I.	p-HBA	Prod	N.I.	2	[43]
139.	Rosaceae	*Malus domestica*	Apple	India	1	Api, Kaem, Myr, Quer, Nar, Pinob, C	CA, ChloA, FA, p-CouA	ElA, GA	N.I.	N.I.	13	[122]
140.	Rosaceae	*Mespilus germanica*	Medlar	Italy	1	Chr, Kaem, Myr, Quer, Rut, Pinoc, EC	CA, ChloA, FA, SinA	GA, VA	N.I.	N.I.	13	[78]
141.	Rosaceae	*Prunus avium*	Cherry blossom	India, Spain	2	Aca, Api, Chr, Tec, Gal, Isor, Kaem, Myr Quer, Quer-3-ME, Isosak, Nar, Pinoc, Pinob, C	CA, CAPE, ChloA, FA, p-CouA	ElA, GA	N.I.	N.I.	24	[51,122]
142.	Rosaceae	*Prunus dulcis* L.	Almond	Italy	1	Chr, Kaem, Myr, Quer, Rut, Pinoc, EC	CA, ChloA, FA, SinA	GA, SyrA, VA	N.I.	N.I.	14	[78]
143.	Rosaceae	*Rosa acicularis*	Wild rose	China	1	Lut, Quer, Hesd	CA, ChloA	GA, p-HBA, SyrA	HGA, Prod	N.I.	10	[43]
144.	Rosaceae	*Rubus chamaemorus*	Clodberry	Finland	1	N.I.	N.I.	p-HBA	3-PPA	N.I.	2	[89]
145.	Rosaceae	*Rubus idaeus*	Raspberry	Czech Republic, Lithuania	2	Chr	CA, ChloA, FA, RosA, t-CA	GA, VA	N.I.	N.I.	8	[65]^,^ [93]
146.	Rutaceae	*Citrus bergamia*	Bergamot	China	1	Api, Chr, Querc	N.I.	GA, GenA, p-HBA	Prod	N.I.	7	[43]
147.	Rutaceae	*Citrus* sp.	Citrus	Algeria, China, Greece, Italy, Portugal, Spain, Turkey, USA	11	Api, Chr, Lut, Gal, Isor, Kaem, Myr, Quer, Rut, Hest, Isosak, Nar, Pinoc, Pinob, CG, Gene	CA, ChloA, FA, o-CouA, p-CouA, t-CA	BenA, GA, GenA, ProA, p-HBA, SyrA, VA	HGA, 3,4-DHPAA, Prod	N.I.	32	[10,33,38,44,46,49,53,98,114,133,153]
148.	Rutaceae	*Citrus* sp., *C. lemon*, *C. limon*, *C. limon Burm*	Lemon blossom, Lemon	Argentina, Bulgaria, China, India, Italy, Portugal, Spain	8	Api, Chr, Lut, Gal, Kaem, Myr, Quer, Rut, Hest, Hesd, Nar, Pinoc, C, EC	CA, ChloA FA, p-CouA, SinA, t-CA	2,3,4-THBA, GA, m-HBA, p-HBA, ProA, SyrA, VA	PAA, DL-β-PLA, Prod	N.I.	30	[43,46,59,78,102,137,154]
149.	Rutaceae	*Citrus* sp., *C. sinensis*, *C. aurantium* L.	Orange, Orange blossom	Austria, Brazil, Bulgaria, China, France, Greece, Italy, Spain, Switzerland, Tunisia	14	Aca, Api, Chr, Chr-2′-ME, Genk, Lut, Tec, Gal, Gal-3-ME, Isor, Kaem, Kaem-8-ME, Myr, Myr-3,7,4′5′-TeME, Quer, Quer-3,7-DME, Quer-3-ME, Rut, Hest, Isosak, Nar, Narg, Pinoc, Pinos, Pinob, Pinob-3-O-ace, Pinob-5-ME, EC, Pinob Chal	3,4-DMCA, CA, CAPE, ChloA, FA, MF, o-CouA, p-CouA, SinA, t-CA	2,3,4-THBA, BenA, ElA, GA, M-4-HBz, MS, ProA, p-HBA, SyrA, VA, VAME	PAA, DL-β-PLA, 5-Phenylpent-4-enoic acid, 2-M-4-VP, 2,3,5-TMP, 2-MBd, Prod	N.I.	57	[43,47,49,51,52,57,59,61,78,97,104,132,154,155]
150.	Salicaceae	*Azara integrifolia*, *A. petiolaris*	Azara	Chile	2	Api, Chr, Lut, Quer, Rut, Pinoc	CA, p-CouA	SyrA	N.I.	AbsA	10	[156]
151.	Salicaceae	*Salix* sp.	Willow Polish, Willow	Poland	2	N.I.	CA, ChloA, FA, p-CouA	BenA, MS, ProA, p-HBA, VA	N.I.	AbsA, KyA	11	[84,157]
152.	Sapindaceae	*Dimocarpus longan*	Longan	China Thailand Malaysia	6	Api, Chr, Lut, Gal Kaem Myr Quer Rut, Nar, Narg, Pinoc, C	3,4-DMCA, CA, ChloA, FA, p-CouA, RosA, t-CA	BenA, ElA, GA, GenA, ProA, p-HBA, ResA, SyrA, VA	Prod	N.I.	29	[43,64,77,135,143]
153.	Sapindaceae	*Guioa semiglauca*	Crow ash	Australia	1	N.I.	ChloA, CA, FA, p-CouA	ElA, GA, SyrA	N.I.	AbsA	8	[58]
154.	Sapindaceae	*Litchi chinensis*	Litchi	China, India	2	Api, Chr, Lut, Gal Kaem Myr Quer Rut, Pinoc, C, EC, Proc	3,4-DMCA, CA, FA, SinA	GA, ProA, p-HBA, VA	4-MPC	N.I.	21	[64,137]
155.	Schisandraceae	*Schisandra chinensis*	Schisandra	China	2	Api, Chr, Lut, Gal, Isor, Kaem, Quer, Rut, Pinoc	3,4-DMCA, CA, FA, IfA, p-CouA, SinA	BenA, GA, ProA, p-HBA, SyrA, VA	N.I.	N.I.	21	[64,96]
156.	Simaroubaceae	*Ailanthus altissima*	Tree of heaven	Italy	1	Api, Chr, Gal, Kaem, Myr, Quer, Gene	CA, ChloA, p-CouA	N.I.	N.I.	N.I.	10	[97]
157.	Solanaceae	*Lycium* sp.	Wolfberry	China	1	Quer, Hesd	CA, ChloA	2,3,4-THBA, GA, GenA, p-HBA	Prod, Van	N.I.	10	[43]
158.	Zingiberaceae	*Zingeber officinale*	Ginger	India	1	Kaem, Quer, Rut, C, EC	CA, ChloA, FA	GA, SyrA, VA	Gin	N.I	12	[137]
159.	Zygophyllaceae	*Zygophyllum album* L.	Zygophyllum	Algeria	1	Quer, Rut	CA, ChloA, p-CouA	GA, VA	Van	N.I	8	[158]

Flavonoids. *Flavone*: acacetin-**Aca**, apigenin-**Api**, baicalin-**Bai**, chrysin-**Chr**, chrysin-2’-methylether-**Chr-2′-ME**, chrysin-6-methylether-**Chr-6-ME**, genkwanin-**Genk**, luteolin-**Lut**, tangeritin-**Tang**, tectochrysin-**Tec**, tricetin-**Tri**, and vitexin-**Vit**; *flavonol*: fisetin-**Fis**, galangin-**Gal**, galangin-5-methylether-**Gal-5-ME**, galangin-3-methylether-**Gal-3-ME**, isorhamnetin-**Isor**, isorhamnetin-4’-diGlc-**isor-4’-diGlc**, isorhamnetin-4’-gentiobioside**-isor-4’-gent**, isorhamnetin-4’-Glc-isor-**4’-Glc**, isorhamnetin-3-Glc-4’-gentiobioside-**isor-3-Glc-4’-gent**, kaempferol-**Kaem**, kaempferol methyl ether-**Kaem-ME**, kaempferol-8-methylether-**Kaem-8-ME**, kaempfeol-7-O-rham**-kaem-7-O-rham**, kaempferol-3-O-(6”-acetyl)-beta-glucopyranoside-**Kaem-3-O-(6”-acetyl)-β-Glucpsde**, kaempferol-3-O-(hexoxyl) robinoside-7-O-rhamnoside-**kaem-3-O-(hexoxyl) rob-7-O-rham**, kaempferol-3-O-(hexoxyl)robinoside-**kaem-3-O-(hexoxyl)rob**, kaempferol-3-O-hexoside-7-O-rhamnoside-**kaem-3-O-hex-7-O-rham**, kaempferol-3-O-robinoside-7-O-rhamnoside-**kaem-3-O-rob-7-O-rham**, kaempferol-3-O-robinoside-**kaem-3-O-rob**, kaempferol-3-diGlc isomer-**kaem-3-diGlc isomer**, kaempferol-3-sophoroside-**kaem-3-soph**, kaempferol-4’-Glc-**kaem-4’-Glc**, kaempferol-3-O-neoh-**kaem-3-O-neoh**, kaempferide-**Kaemf**, morin-**Mor**, myricetin-**Myr**, myricetin-3-methylether-**Myr-3-ME**, myricetin-3,7,4′5′-tetramethylether**-Myr-3,7,4′5′-TeME**, quercetin-**Quer**, quercetin-3,3-dimethylether-**Quer-3,3-DME**, quercetin-3,7-dimethylether-**Quer-3,7-DME**, quercetin-3-methylether-**Quer-3-ME**, quercetin-3,3’,4’-triGlc-**Quer-3,3’,4’-triGlc**, quercetin-3,4’-diGlc-**Quer-3,4’-diGlc**, quercetin-3-sophoroside-**Quer-3-soph**, quercetin-3-O-hex (1→2) hex-**quer-3-O-hex (1→2) hex**, quercetin diglycoside-**Quer-diGlc**, quercetin rhamnoside-**Quer-rham**, quercetin-7,3’-dimethyl ether-**Quer-7,3’-DME**, quercitrin-**Querc**, rhamnetin-**Rham**, and rutin-**Rut**; *flavonone*: alpinetin-**Alp**, eriodictoyl-**Erio**, hesperitin-**Hest**, hesperidin-**Hesd**, isosakuranetin-**Isosak**, naringenin-**Nar**, naringen-methylether-**Nar-ME,** naringin-**Narg**, pinocembrin-**Pinoc**, pinostrobin-**Pinos**, sakuranetin-**Sak**; *flavononol* pinobanksin-**Pinob**, pinobanksin-3-O-acetate-**Pinob-3-O-ace**, pinobanksin-3-O-butyrate-**Pinob-3-O-butyr**, pinobanksin-3-O-pentenoate-**pinob-3-O-pent**, pinobanksin-3-O-propionate-**pinob-3-O-prop**, pinobanksin-5-methyl ether-**Pinob-5-ME**, and taxifolin-**Tax**; *flavan-3-ol*: catechin-**C**, catechin gallate-**CG**, epicatechin-**EC**, epigallocatechin-**EGC**, epigallocatechin gallate-**EGCG**, gallocatechin-**GC**, and gallocatechin gallate-**GCG**; anthocyanidine: procyanidine dimer-**Proc**; *isoflavonoids*: calycosin-**Cal**, daidzein-Dai, formononetin-**Form**, genistein-**Gene**, genistin-**Geni**, ononin-**Ono**, and calycosin 7-O-β-D-glucoside-**cal 7-O-β-D-gluc**; *aurone*: Leptosin-**Leptosin**; *chalcone*: pinobanksin chalcone-**Pinob Chal**; hydroxycinnamic acid and derivatives (**HCAD**): 3,4 dimethoxycinnamic acid-**3,4-DMCA**, caffeic acid-**CA**, caffeic acid benzyl ester-**CABE**, caffeic acid dimethylallyl ester-**CADAE**, caffeic acid isoprenyl ester-**CAIPE**, caffeic acid phenethyl ester-**CAPE**, chlorogenic acid-**ChloA**, chrytochlorogenic acid-**CChloA**, ferulic acid-**FA**, isoferulic acid-**IfA**, m-coumaric acid-**m-CouA**, m-methoxycinnamic acid-**m-MCA**, methyl ferulate-**MF**, o-coumaric acid-**o-CouA**, p-coumaric acid-**p-CouA**, p-methoxycinnamic acid-**p-MCA**, rosmarinic acid-**RosA**, sinapic acid-**SinA**, t-cinnamic acid-**t-CA**, and trans-p-coumaric acid methyl ester-**t-p-CouAME**; hydroxybenzoic acids and derivatives (**HBAD**): 2,3,4 trihydrobenzoic acid-2,3,4-THBA, 3,4,5-trimethoxybenzoic acid-3,4,5-TMBA, benzoic acid-BA, cuminic acid-CuA, ellagic acid-ElA, gallic acid-GA, gentisic acid-**GenA**, leptosperin-**Lepp**, methyl 4-hydroxybenzoate-**M-4-HBz**, methyl syringate-**MS**, m-hydroxybenzoic acid-**m-HBA**, o-anisic acid-**OAA**, p-anisic acid-**PAA**, penta-O-galloyl-β-D-glucose (PGG)-**PGG**, protocatechuic acid-**proA**, p-hydroxybenzoic acid-**p-HBA**, resorcylic acid-**ResA**, salicylic acid-**SalA**, syringic acid-**SyrA**, vanillic acid-**VA**, and vanillic acid methyl ester-**VAME;** other phenolics/miscellaneous: 3,4-dihydroxyphenylacetic acid-**3,4-DHPAA**, homogentisic acid-**HGA**, homovanillic acid-**HVA**, mandelic acid-**MandA**, phenylacetic acid-**PAA**, p-hydroxyphenylacetic acid -**p-HPAA**, 4-methoxyphenyllactic acid-**4-mPLA**, DL-p hydroxyphenyllactic acid-**DL-p-HPLA**, DL-β-phenyllactic acid-**DL-β-PLA**, 3-phenyl propionic acid-**3-PPA**, phloretic acid-**PhlA**, 2-Methoxy-4-vinylphenol-**2-M-4-VP**, 2,3,5 trimethyl phenol-**2,3,5-TMP**, 3,4,5-trimethylphenol-**3,4,5-TMP**, 4-methylpyrocatechol-**4-MPC**, 2-methylbenzaldehyde-**2-MBd**, p-anisaldehyde-**p-And**, protocatechualdehyde-**Prod**, vanillin-**Van**, 2’-hydroxyacetophenone-**2’-HAPo**, 2’-methoxyacetophenone-**2’-MAPo**, gingerol-**Gin**, 5,7-dimethoxycoumarin-**5,7-DMCoum**, emodin-**Emo**, nor-β-lapachone-**n-β-L**, resveratrol-**Resv**, and dibenzyl oxalate-**DBZO;** non-phenolics: 2-methylbenzofuran-**2-MBF**, 5-methylfuran-3-carboxylic Acid-**5-MF-3-CA**, absiscic acid-**AbsA**, kojic acid-**KojA**, kynurenic acid-**KyA**, lepteridine-**Leptd**, lumichrome-**Lum**, phenylalanine-**PhAn**, and tyrosin-**Tyr, N.I-No Information**.

**Table 2:** Flavones reported in monofloral honeys (see Figure 7 for general structure).

R5	R6	R7	R8	R2′	R3′	R4′	R5′	R6′	Name	Code	CAS No.	No. of Honeys
-O-H	-H	-O-Me	-H	-H	-H	-O-Me	-H	-H	Acacetin	**1**	480-44-4	15
-O-H	-H	-O-H	-H	-H	-H	-O-H	-H	-H	Apigenin	**2**	520-36-5	74
-O-H	-H	-O-Glc	-H	-H	-H	-H	-H	-H	Baicalin	**3**	21967-41-9	6
-O-H	-H	-O-H	-H	-H	-H	-H	-H	-H	Chrysin	**4**	480-40-0	83
-O-H	-H	-O-H	-H	-O-Me	-H	-O-H	-H	-H	Chrysin-2′-methylether	**5**	10458-35-2	6
-O-H	-O-Me	-O-H	-H	-H	-H	-H	-H	-O-H	Chrysin-6-methylether	**6**	480-11-5	5
-O-H	-H	-O-Me	-H	-H	-H	-O-H	-H	-H	Genkwanin	**7**	437-64-9	6
-O-H	-H	-O-H	-H	-H	-O-H	-O-H	-H	-H	Luteolin	**8**	491-70-3	69
-O-Me	-O-Me	-O-Me	-O-Me	-H	-H	-O-Me	-H	-H	Tangerin	**9**	481-53-8	1
-O-H	-H	-O-Me	-H	-H	-H	-H	-H	-H	Tectochrysin	**10**	520-28-5	16
-O-H	-H	-O-H	-H	-H	-O-H	-O-H	-O-H	-H	Tricetin	**11**	520-31-0	5
-O-H	-H	-O-H	-Glc	-H	-H	-O-H	-H	-H	Vitexin	**12**	3681-93-4	10

Legend: -H—hydride, -O-H—hydroxide, -O-Me—methoxide, and -Glc—glucoside.

**Table 3:** Flavonols reported in monofloral honeys (see Figure 8 for general structure).

R3	R5	R7	R8	R2′	R3′	R4′	R5′	Name	Code	CAS No.	No. of Honeys
-O-H	-H	-O-H	-H	-H	-O-H	-O-H	-H	Fisetin	**13**	528-48-3	6
-O-H	-O-H	-O-H	-H	-H	-H	-H	-H	Galangin	**14**	548-83-4	66
-O-H	-O-Me	-O-H	-H	-H	-H	-H	-H	Galangin-5-methyl ether	**15**	104594-69-6	5
-O-Me	-O-H	-O-H	-H	-H	-H	-H	-H	Galangin-3-methyl ether	**16**	6665-74-3	3
-O-H	-O-H	-O-H	-H	-H	-O-Me	-O-H	-H	Isorhamnetin	**17**	480-19-3	43
-O-H	-O-H	-O-H	-H	-H	-O-Me	-O-diGlc	-H	Isorhamnetin-4′-diglucoside	**18**	N.I.	2
-O-H	-O-H	-O-H	-H	-H	-O-Me	-O-Gen	-H	Isorhamnetin-4′—gentiobioside	**19**	N.I.	2
-O-H	-O-H	-O-H	-H	-H	-O-Me	-O-Glc	-H	Isorhamnetin-4′-Glc	**20**	N.I.	2
-O-Glc	-O-H	-O-H	-H	-H	-O-Me	-O-Gen	-H	isorhamnetin-3-Glc-4’-gentiobioside	**21**	N.I.	2
-O-H	-O-H	-O-H	-H	-H	-H	-O-H	-H	Kaempferol	**22**	520-18-3	89
-O-H	-O-H	-O-H	-H	-H	-H	-O-H	-H	Kaempferol-*-methylether	**23**	N.I.	3
-O-H	-O-H	-O-H	-O-Me	-H	-H	-O-H	-H	Kaempferol-8-methylether	**24**	571-74-4	22
-O-H	-O-H	-O-Rham	-H	-H	-H	-O-H	-H	Kaempferol-7-O-rhamnoside	**25**	N.I.	6
-O-H	-O-H	-O-H	-H	-H	-H	-O-H	-H	Kaempferol3-O-(6”-acetyl)-beta-glucopyranoside	**26**	N.I.	3
-O-(hexoxyl) rob	-O-H	-O-Rham	-H	-H	-H	-O-H	-H	Kaempferol-3-O-(hexoxyl) robinoside-7-O-rhamnoside	**27**	N.I.	1
-O-(hexoxyl) rob	-O-H	-O-H	-H	-H	-H	-O-H	-H	Kaempferol-3-O-(hexoxyl)robinoside	**28**	N.I.	1
-O-hex	-O-H	-O-Rham	-H	-H	-H	-O-H	-H	kaempferol-3-O-hexoside-7-O-rhamnoside	**29**	N.I.	1
-O-rob	-O-H	-O-Rham	-H	-H	-H	-O-H	-H	Kaempferol-3-O-robinoside-7-O-rhamnoside	**30**	N.I.	1
-O-Rob	-O-H	-O-H	-H	-H	-H	-O-H	-H	Kaempferol-3-O-robinoside	**31**	N.I.	1
-O-diGlc	-O-H	-O-H	-H	-H	-H	-O-H	-H	Kaempferol-3-diGlc isomer	**32**	N.I.	2
-O-Soph	-O-H	-O-H	-H	-H	-H	-O-H	-H	Kaempferol-3-sophoroside	**33**	N.I.	2
-O-H	-O-H	-O-H	-H	-H	-H	-O-Glc	-H	Kaempferol-4’-Glc	**34**	N.I.	2
-O-Neoh	-O-H	-O-H	-H	-H	-H	-O-H	-H	Kaempferol-3-O- neohespeidoside	**35**	N.I.	3
-O-H	-O-H	-O-H	-H	-H	-H	-O-Me	-H	Kaempferide	**36**	491-54-3	7
-O-H	-O-H	-O-H	-H	-O-H	-H	-O-H	-H	Morin	**37**	480-16-0	7
-O-H	-O-H	-O-H	-H	-H	-O-H	-O-H	-O-H	Myricetin	**38**	529-44-2	54
-O-Me	-O-H	-O-H	-H	-H	-O-H	-O-H	-O-H	Myricetin-3-methyl ether	**39**	1486-67-5	1
-O-Me	-O-H	-O-Me	-H	-H	-O-Me	-O-Me	-O-H	Myricetin-3,7,4′5′-tetramethyl ether	**40**	14290-57-4	6
-O-H	-O-H	-O-H	-H	-H	-O-H	-O-H	-H	Quercetin	**41**	117-39-5	102
-O-Me	-O-H	-O-H	-H	-H	-O-Me	-O-H	-H	Quercetin-3,3-dimethyl ether	**42**	4382-17-6	8
-O-Me	-O-H	-O-Me	-H	-H	-O-H	-O-H	-H	Quercetin-3,7-dimethyl ether	**43**	2068-02-2	10
-O-Me	-O-H	-O-H	-H	-H	-O-H	-O-H	-H	Quercetin-3-methyl ether	**44**	1486-70-0	15
-O-Glc	-O-H	-O-H	-H	-H	-O-Glc	-O-Glc	-H	Quercetin-3,3’,4’-triGlc	**45**	N.I.	2
-O-Glc	-O-H	-O-H	-H	-H	-O-H	-O-Glc	-H	Quercetin-3,4’-diGlc	**46**	N.I.	2
-O-Soph	-O-H	-O-H	-H	-H	-O-H	-O-H	-H	Quercetin-3-sophoroside	**47**	N.I.	2
-O-hex (1→2) hex	-O-H	-O-H	-H	-H	-O-H	-O-H	-H	Quercetin-3-O-hex (1→2) hex	**48**	N.I.	2
N/A	N/A	N/A	N/A	N/A	N/A	N/A	N/A	Quercitin diglycoside	**49**	N.I.	1
N/A	N/A	N/A	N/A	N/A	N/A	N/A	N/A	Quercitin rhamnoside	**50**	N.I.	7
-O-H	-O-H	-O-Me	-H	-H	-O-Me	-O-H	-H	Quercetin-7,3’ dimethyl ether	**51**	552-54-5	1
-O-Rham	-O-H	-O-H	-H	-H	-O-H	-O-H	-H	Quercitrin	**52**	522-12-3	16
-O-H	-O-H	-O-Me	-H	-H	-O-H	-O-H	-H	Rhamnetin	**53**	90-19-7	9
-O-Rut	-O-H	-O-H	-H	-H	-O-H	-O-H	-H	Rutin	**54**	153-18-4	58

Legend: -H—hydride, -O-H—hydroxide, -O-Me—methoxide, - Glc—glucoside, -O-Gen—gentiobioside, -O-Rham—rhamnoside, -O-Gen—gentiobioside, -O-(hexoxyl)rob—(hexoxyl) robinoside, -O-Soph—sophoroside, -O-Neoh—neohespeidoside, -O-hex—hexoside, -O-Rut—rutoside; N.I.—no information, and N/A—information not provided.

**Table 4:** Flavanones reported in monofloral honeys (see Figure 9 for general structure).

R5	R7	R3′	R4′	Name	Code	CAS No.	No. of Honeys
-O-Me	-O-H	-H	-H	Alpinetin	**55**	36052-37-6	1
-O-H	-O-H	-O-H	-O-H	Eriodictyol	**56**	552-58-9	5
-O-H	-O-H	-O-H	-O-Me	Hesperitin	**57**	520-33-2	49
-O-H	-O-Rut	-O-H	-O-Me	Hesperidin	**58**	520-26-3	14
-O-H	-O-H	-H	-O-Me	Isosakuranetin	**59**	480-43-3	18
-O-H	-O-H	-H	-O-H	Naringenin	**60**	67604-48-2	54
-O-H	-O-H	-H	-O-H	Naringenin-?-methyl ether	**61**	N.I.	1
-O-H	-O-Rut	-H	-O-H	Naringin	**62**	10236-47-2	9
-O-H	-O-H	-H	-H	Pinocembrin	**63**	480-39-7	64
-O-H	-O-Me	-H	-H	Pinostrobi	**64**	480-37-5	9
-O-H	-O-Me	-H	-O-H	Sakuranetin	**65**	2957-21-3	8

Legend: -H—hydride, -O-H—hydroxide, -O-Me—methoxide, -O-Rut—rutoside, -O-Prop—propionate, -O-But—butyrate, -O-Ace—acetate, -O-Pent—pentenoate, -O-Gall—gallate, N.I.—no information, ?—substituent location not indicated

**Table 5:** Flavanonols reported in monofloral honeys (see Figure 10 for general structure).

R3	R5	R3′	R4′	Name	Code	CAS No.	No. of Honeys
-O-H	-O-H	-H	-H	Pinobanksin	**66**	548-82-3	49
-O-Ace	-O-H	-H	-H	Pinobanksin-3-O-acetate	**67**	52117-69-8	5
-O-But	-O-H	-H	-H	Pinobanksin-3-O-butyrate	**68**	126394-71-6	1
-O-Pent	-O-H	-H	-H	Pinobanksin-3-O-pentenoate	**69**	N.I.	1
-O-Prop	-O-H	-H	-H	Pinobanksin-3-O-propionate	**70**	126394-70-5	1
-O-H	-O-Me	-H	-H	Pinobanksin-5-methyl ether	**71**	87620-04-0	7
-O-H	-O-H	-O-H	-O-H	Taxifolin	**72**	480-18-2	8

Legend: -H—hydride, -O-H—hydroxide, -O-Me—methoxide, -O-Prop—propionate, -O-But—butyrate, -O-Ace—acetate, -O-Pent—pentenoate, -O-Gall—gallate, and N.I.—no information.

**Table 6:** Flavan-3-ols reported in monofloral honeys (see Figure 1 for general structure).

R3	R5′	Name	Code	CAS No.	No. of Honeys
-O-H	-H	Catechin	**73**	154-23-4	29
-O-Gall	-H	Catechin gallate	**74**	130405-40-2	12
-O-H	-H	Epicatechin	**75**	490-46-0	24
-O-H	-O-H	Epigallocatechin	**76**	970-74-1	5
-O-Gall	-O-H	Epigallocatechin gallate	**77**	989-51-5	1
-O-H	-O-H	Gallocatechin	**78**	970-73-0	9
-O-Gall	-O-H	Gallocatechin gallate	**79**	4233-96-9	1
N/A	N/A	‘Procyanidin’ ^#^	**80**	4852-22-6	1

Legend: -H—hydride, -O-H—hydroxide, -O-Gall—gallate, and ^#^ no further structural information provided.

**Table 7:** Isoflavonoids reported in honeys (see Figure 12 for general structure).

R5	R7	R3′	R4′	Name	Code	CAS No.	No. of Honeys
-H	-O-H	-O-H	-O-Me	Calycosin	**81**	20575-57-9	3
-H	-O-Glc	-O-H	-O-Me	Calycosin-7-O-β-D-glucoside	**82**	20633-67-4	3
-H	-O-H	-O-H	-H	Daidzein	**83**	486-66-8	5
-H	-O-H	-H	-O-Me	Formononetin	**84**	485-72-3	8
-O-H	-O-H	-H	-O-H	Genistein	**85**	446-72-0	23
-O-H	-O-Glc	-H	-O-H	Genistin	**86**	529-59-9	2
-H	-O-Glc	-H	-O-Me	Ononin	**87**	486-62-4	6

Legend: -H—hydride, -O-H—hydroxide, -O-Me—methoxide, and -O-Glc—glucoside.

**Table 8:** Aurones and chalcones reported in monofloral honeys.

Subclass	Name	Code	CAS No.	No. of Honeys
Aurone	Leptosin	**88**	486-23-7	5
Chalcone	Pinobanksin chalcone	**89**	N.I.	6

**Table 9:** Hydroxycinnamic acid and its derivatives reported in monofloral honeys (see Figure 14 for general structure).

OR1	R5	R6	R7	R8	Name	Code	CAS No.	No of Honeys
-H	-H	-O-Me	-O-Me	-H	3,4 Dimethoxycinnamic acid	**90**	2316-26-9	11
-H	-H	-O-H	-O-H	-H	Caffeic acid	**91**	331-39-5	117
-benzyl	-H	-O-H	-O-H	-H	Caffeic acid benzyl ester	**92**	107843-77-6	1
-dimethylallyl	-H	-O-H	-O-H	-H	Caffeic acid dimethylallyl ester	**93**	100884-13-7	8
-isoprenyl	-H	-O-H	-O-H	-H	Caffeic acid isoprenyl ester	**94**	N.I.	2
-phenyl	-H	-O-H	-O-H	-H	Caffeic acid phenethyl ester	**95**	104594-70-9	17
-QA (3)	-H	-O-H	-O-H	-H	Chlorogenic acid	**96**	327-97-9	85
-QA (4)	-H	-O-H	-O-H	-H	Cryptochlorogenic acid	**97**	905-99-7	7
-H	-H	-O-Me	-O-H	-H	Ferulic acid	**98**	537-98-4	102
-H	-H	-O-H	-O-Me	-H	Isoferulic acid	**99**	537-73-5	12
-H	-H	-O-H	-H	-H	m-Coumaric acid	**100**	14755-02-3	10
-H	-H	-O-Me	-H	-H	m-Methoxycinnamic acid	**101**	6099-04-3	1
-Me	-H	-O-Me	-O-H	-H	Methyl ferulate	**102**	2309-07-1	3
-H	-O-H	-H	-H	-H	o-Coumaric acid	**103**	614-60-8	23
-H	-H	-H	-O-H	-H	p-Coumaric acid	**104**	501-98-4	103
-H	-H	-H	-O-Me	-H	p-Methoxycinnamic acid	**105**	830-09-1	2
-3,4-DHPLA	-H	-O-H	-O-H	-H	Rosmarinic acid	**106**	20283-92-5	25
-H	-H	-O-Me	-O-H	-Me	Sinapic acid	**107**	530-59-6	27
-H	-H	-H	-H	-H	t-Cinnamic acid	**108**	140-10-3	56
-Me	-H	-H	-O-H	-H	trans-p-Coumaric acid methyl ester	**109**	19367-38-5	1

Legend: -H—hydride, -O-H—hydroxide, -O-Me—methoxide, -Me—methyl, QA—quinic acid, HCAD—hydroxycinnamic acid and derivatives, 3,4-DHPLA—3,4-dihydroxyphenyl lactic acid, and N.I.—no information.

**Table 10:** Hydroxybenzoic acid and its derivatives reported in monofloral honeys (see Figure 15 for general structure, see Figure 16 for ellagic acid and penta-O-galloyl-β-D-glucose (PGG) structure).

OR1	R3	R4	R5	R6	Name	Code	CAS No.	No. of Honeys
-H	-O-H	-O-H	-O-H	-H	2,3,4 Trihydrobenzoic acid	**110**	610-02-6	12
-H	-H	-O-Me	-O-Me	-O-Me	3,4,5-Trimethoxybenzoic acid	**111**	118-41-2	4
-H	-H	-H	-H	-H	Benzoic acid	**112**	65-85-0	43
-H	-H	-H	-Isopropyl	-H	Cuminic acid	**113**	536-66-3	4
See Figure 16					Ellagic acid	**114**	476-66-4	45
-H	-H	-O-H	-O-H	-O-H	Gallic acid	**115**	149-91-7	105
-H	-O-H	-H	-H	-O-H	Gentisic acid	**116**	490-79-9	35
-Me	-H	-O-Me	-O-gent	-O-Me	Leptosperin	**117**	N.I	2
-Me	-H	-H	-O-H	-H	Methyl 4-hydroxybenzoate	**118**	99-76-3	6
-Me	-H	-O-Me	-O-H	-O-Me	Methyl syringate	**119**	884-35-5	23
-H	-H	-O-H	-H	-H	m-Hydroxybenzoic acid	**120**	99-06-9	14
-H	-O-Me	-H	-H	-H	o-Anisic acid	**121**	579-75-9	6
-H	-H	-H	-O-Me	-H	p-Anisic acid	**122**	100-09-4	6
See Figure 16					Penta-O-galloyl-β-D-glucose (PGG)	**123**	14937-32-7	1
-H	-H	-O-H	-O-H	-H	Protocatechuic acid	**124**	99-50-3	57
-H	-H	-H	-O-H	-H	p-Hydroxybenzoic acid	**125**	99-96-7	79
-H	-O-H	-O-H	-H	-H	Resorcylic acid	**126**	303-38-8	13
-H	-O-H	-H	-H	-H	Salicylic acid	**127**	69-72-7	20
-H	-H	-O-Me	-O-H	-O-Me	Syringic acid	**128**	530-57-4	85
-H	-H	-O-Me	-O-H	-H	Vanillic acid	**129**	121-34-6	66
-Me	-H	-O-Me	-O-H	-H	Vanillic acid methyl ester	**130**	3943-74-6	7

Legend: -H—hydride, -O-H—hydroxide, -O-Me—methoxide, -Me—methyl, -O-Gent—gentibioside, and N.I.—no information.

**Table 11:** Hydroxyphenylacetic acid and derivatives (HPAAD) reported in monofloral honeys (see Figure 17 for general structure).

R2b	R5	R6	R7	Name	Code	CAS No.	No. of Honeys
-H	-H	-O-H	-O-H	3,4-Dihydroxyphenylacetic acid	**131**	102-32-9	10
-H	-O-H	-H	-O-H	Homogentisic acid	**132**	451-13-8	24
-H	-O-Me	-O-H	-H	Homovanillic acid	**133**	306-08-1	4
-O-H	-H	-H	-H	Mandelic acid	**134**	90-64-2	3
-H	-H	-H	-H	Phenylacetic acid	**135**	103-82-2	17
-H	-H	-O-H	-H	p-Hydroxyphenylacetic acid	**136**	156-38-7	17

Legend: -H—hydride, -O-H—hydroxide, -O-Me—methoxide, and -Me—methyl.

**Table 12:** Hydroxyphenyllactic acid and derivatives (HPLAD) and hydroxyphenylpropanoic acid and derivatives (HPPAD) reported in monofloral honeys (see Figure 18 for general structure).

R2b	R7	Subclass	Name	Code	CAS No.	No. of Honeys
-O-H	-O-H	HPLAD	DL-p hydroxyphenyllactic acid	**137**	306-23-0	4
-O-H	-O-Me	HPLAD	4-Methoxyphenyllactic acid	**138**	N.I.	7
-O-H	-H	HPLAD	DL-β-Phenyllactic acid	**139**	828-01-3	23
-H	-H	HPPAD	3-Phenyl propionic acid	**140**	501-52-0	13
-H	-O-H	HPPAD	Phloretic acid	**141**	501-97-3	3

Legend: -H—hydride, -O-H—hydroxide, -O-Me—methoxide, and N.I.—no information.

**Table 13:** Hydroxyphenylpentanoic acid (HPPeA) reported in monofloral honeys.

Subclass	Name	Code	CAS No.	No. of Honeys
HPPeA	5-Phenylpent-4-enoic acid	**142**	306-23-0	5

**Table 14:** Alkylmethoxyphenol, alkylphenols, hydroxybenzaldehydes, hydroxybenzoketones and hydroxyphenylketone reported in monofloral honeys (see Figure 20 for general structure).

**R1**	**R2**	**R3**	**R4**	**R5**	**Subclass**	**Name**	**Code**	**CAS No.**	**No. of Honeys**
-O-H	-O-Me	-H	-vinyl	-H	AMPh	2-Methoxy-4-vinylphenol	**143**	7786-61-0	6
-O-H	-Me	-Me	-H	-Me	APh	2,3,5-Trimethyl phenol	**144**	697-82-5	3
-O-H	-H	-Me	-Me	-Me	APh	3,4,5-Trimethyl phenol	**145**	527-54-8	3
-O-H	-O-H	-H	-H	-Me	APh	4-Methylpyrocatechol	**146**	452-86-8	5
-C(=O)H	-Me	-H	-H	-H	HBzd	2-Methylbenzaldehyde	**147**	529-20-4	6
-C(=O)H	-H	-H	-O-Me	-H	HBzd	p-Anisaldehyde	**148**	123-11-5	2
-C(=O)H	-H	-O-H	-O-H	-H	HBzd	Protocatechualdehyde	**149**	139-85-5	40
-C(=O)H	-H	-O-Me	-O-H	-H	HBzd	Vanillin	**150**	121-33-5	18
-ethanone	-H	-O-Me	-H	-H	HAPhn	1-(3-Methoxy-phenyl)-ethanone	**151**	586-37-8	1
- ethanone	-O-H	-H	-H	-H	HAPhn	2’-Hydroxyacetophenone	**152**	118-93-4	1
- ethanone	-O-Me	-H	-H	-H	HAPhn	2’-Methoxyacetophenone	**153**	579-74-8	3
-5-hydroxydeca-3-one	-H	-O-Me	-O-H	-H	Guaiacol	Gingerol	**154**	23513-14-6	1
- butan-1-one	-NH_2_	-H	-H	-H	Others	1-(2-Aminophenyl)butan-1-one	**155**	2034-40-4	1
-3-hydroxy-penta-1,4-dione	-O-Me	-H	-H	-H	Others	3-Hydroxy-1-(2-methoxyphenyl)penta-1,4-dione	**156**	N.I.	1

Legend: -H—hydride, -O-H—hydroxide, -O-Me—methoxide, -C(=O) H—aldehyde group, NH_2—_amino group, N.I.—no information., AMPh—alkylmethoxyphenol, APh—alkylphenol, HBzd—hydroxy-benzaldehydes, and HAPhn—hydroxyacetophenone.

**Table 15:** Hydroxycoumarin, anthraquinone, naphthoquinone, stilbenes and benzyl oxalate ester reported in monofloral honeys.

Subclass	Name	Code	CAS No.	No. of Honeys
Hydroxycoumarin	5,7-Dimethoxycoumarin	**157**	487-06-9	5
Anthraquinone	Emodin	**158**	518-82-1	1
Naphthoquinone	Nor-β-lapachone	**159**	52436-88-1	1
Stilbenes	Resveratrol	**160**	501-36-0	1
Oxalate ester	Dibenzyl oxalate	**161**	7579-36-4	5

**Table 16:** Non-phenolic compounds reported in monofloral honeys.

Subclass	Name	Code	CAS No.	No. of Honeys
Non-Phenolics	2-Methylbenzofuran	**162**	4265-25-2	1
Non-Phenolics	5-Methylfuran-3-carboxylic acid	**163**	21984-93-0	3
Non-Phenolics	Absiscic acid	**164**	21293-29-8	36
Non-Phenolics	Kojic acid	**165**	501-30-4	3
Non-Phenolics	Kynurenic acid	**166**	492-27-3	4
Non-Phenolics	Lepteridine	**167**	N.I.	6
Non-Phenolics	Lumichrome	**168**	1086-80-2	6
Non-Phenolics	Phenylalanine	**169**	63-91-2	5
Non-Phenolics	Tyrosin	**170**	556-03-6	5

Legend: N.I.—no information.

**Table 17:** Analytical methods used in phenolic compound analysis for monofloral honeys.

Method	No. of Reports
Fluorescence Spectroscopy	1
GC–MS	6
HPLC (UV, UV–UV, UV–Vis, DAD/PDA, DAD–UV, ECD, ECD–UV)	373
HPLC–DAD and LC–MS	23
HPLC–DAD and LC–MS and GC–MS	3
HPTLC	6
LC–MS	136
N.I. *	8
Total	556

* No information available.

## Data Availability

No new data were created or analyzed in this study. Data sharing is not applicable to this article.
